# Ruthenium(IV) Complexes as Potential Inhibitors of Bacterial Biofilm Formation

**DOI:** 10.3390/molecules25214938

**Published:** 2020-10-26

**Authors:** Agnieszka Jabłońska-Wawrzycka, Patrycja Rogala, Grzegorz Czerwonka, Sławomir Michałkiewicz, Maciej Hodorowicz, Paweł Kowalczyk

**Affiliations:** 1 Institute of Chemistry, Jan Kochanowski University in Kielce, 7 Uniwersytecka Str., 25-406 Kielce, Poland; patrycja.rogala@ujk.edu.pl (P.R.); slawomir.michalkiewicz@ujk.edu.pl (S.M.); 2 Institute of Biology, Jan Kochanowski University in Kielce, 7 Uniwersytecka Str., 25-406 Kielce, Poland; grzegorz.czerwonka@ujk.edu.pl; 3 Faculty of Chemistry, Jagiellonian University, 2 Gronostajowa Str., 30-387 Kraków, Poland; hodorowm@chemia.uj.edu.pl; 4 Department of Animal Nutrition, The Kielanowski Institute of Animal Physiology and Nutrition, Polish Academy of Sciences, 3 Instytucka Str., 05-110 Jabłonna, Poland; p.kowalczyk@ifzz.pl

**Keywords:** ruthenium, structural and spectroscopic studies, electrochemistry, X-ray diffraction, antibacterial and antibiofilm activity

## Abstract

With increasing antimicrobial resistance there is an urgent need for new strategies to control harmful biofilms. In this study, we have investigated the possibility of utilizing ruthenium(IV) complexes (H_3_O)_2_(HL1)_2_[RuCl_6_]·2Cl·2EtOH (**1**) and [RuCl_4_(CH_3_CN)_2_](L^3^2)·H_2_O (**2**) (where L1-2-hydroxymethylbenzimadazole, L^3^2-1,4-dihydroquinoxaline-2,3-dione) as effective inhibitors for biofilms formation. The biological activities of the compounds were explored using *E. coli*, *S. aureus*, *P. aeruginosa* PAO1, and *P. aeruginosa* LES B58. The new chloride ruthenium complexes were characterized by single-crystal X-ray diffraction analysis, Hirshfeld surface analysis, FT-IR, UV-Vis, magnetic and electrochemical (CV, DPV) measurements, and solution conductivity. In the obtained complexes, the ruthenium(IV) ions possess an octahedral environment. The intermolecular classical and rare weak hydrogen bonds, and π···π stacking interactions significantly contribute to structure stabilization, leading to the formation of a supramolecular assembly. The microbiological tests have shown complex **1** exhibited a slightly higher anti-biofilm activity than that of compound **2**. Interestingly, electrochemical studies have allowed us to determine the relationship between the oxidizing properties of complexes and their biological activity. Probably the mechanism of action of **1** and **2** is associated with generating a cellular response similar to oxidative stress in bacterial cells.

## 1. Introduction

The increase in microbial resistance to antibiotics is a global public health problem that threatens the successful treatment of infectious diseases. Combatting this threat is a high priority not only for the European Medicines Agency but also for the World Health Organization. In 2017, a group of international experts led by the WHO published a list of 12 drug-resistant bacteria that require prompt action from the scientific research community to develop new antibiotics to treat these microorganisms [1]. This global list clearly divides pathogens into three priority tiers, namely, critical, high, and medium, depending on the degree of health risk and the level of urgency for the antibiotics needed. Scientists have identified a multi-resistant strain of *Pseudomonas aeruginosa*—a gram-negative bacterium that has the ability to survive in different environments—as the most dangerous bacterium. *P. aeruginosa* infections are involved in several human diseases such as cystic fibrosis, meningitis, and septicaemia. The severe infections caused by this strain contribute to high mortality rates, mostly in hospitalized patients [2,3,4,5]. It is worth noting that antibiotic resistance and thus failures in the treatment of infections are mainly related to the mechanism of pathogenicity of microorganisms, which is the ability to form biofilms [6,7,8,9]. Generally, it is estimated that approximately 80% of all bacterial infections are associated with biofilm formation [6]. The structure of biofilms makes the bacterial cells that build them nearly 1000 times less sensitive to toxic substances (antibiotics, surfactants, and disinfectants) than their planktonic counterparts [7,8]. Moreover, conventional antibiotic therapy is able to eliminate only planktonic cells [7,8]. Studies on improving the treatment of bacterial biofilm infections are still currently being developed. In recent years, there has been an increased interest in the use of coordination complexes of transition metals such as silver, copper, gallium, zinc, cobalt, nickel, and ruthenium as anti-biofilm agents [10,11,12,13,14,15]. In our previous studies, we have reported evaluation results of the anti-biofilm activity of the obtained ruthenium complexes in different oxidation states. To the best of our knowledge, no previous research on the anti-biofilm activity of high-valent ruthenium complexes against *P. aeruginosa* has been investigated. So far, our studies have focused on ruthenium complexes that contain heterocyclic alcohols and carboxylic acids andpossess moderate anti-biofilm activity. Among the tested compounds, the best activity was observed for the chloride Ru(IV) complex in which the protonated ligand acted as a counter ion [16]. Weaker activity was determined for the ruthenium complexes in the +III and +IV oxidation states with N,O-donor ligands [17].

In this study, we have extended the scope of our research, and some efforts have been made to modify the composition of the complexes. These modifications were intended to increase the biological activity of the compounds by introducing heterocyclic alcohols and carboxylic acids in protonated form. Also, Keppler and colleagues have observed significant biological activity of chloride ruthenium complexes (KP1019, NAMI-A) [18,19]. We used 2-hydroxymethylbenzimidazole (L1) and 3-hydroxy-2-quinoxalinecarboxylic acid (L^1^2 commercial) containing privileged structures to achieve this effect. Accordingly, the aim of this work was to investigate the possibility of utilizing Ru(IV) complexes as effective inhibitors for bacterial biofilms of *Pseudomonas aeruginosa* PAO1 (laboratory strain) and *Pseudomonas aeruginosa* LES B58 (clinical strain). This choice resulted from the fact that *P. aeruginosa* was classified as critical, multi-resistant strain. The commonly used *Escherichia coli* ATCC 8739 and *Staphylococcus aureus* ATCC 6538P were also tested. In this paper, we studied the following aspects: (1) to carry out the syntheses of new chloride Ru(IV) complexes and describe their crystal structures and physical-chemical properties; (2) to investigate of the interactions between molecules in crystals; (3) to study the redox properties of the Ru(IV) complexes (by CV and DPV methods); (4) to gain information on the inhibition of bacterial growth and biofilm formation in the tested strains caused by ruthenium complexes; (5) to investigate oxidative DNA damage using the formamidopyrimidine-DNA glycosylase (Fpg); (6) to evaluate the regularity between electrochemical properties and biological activity.

## 2. Results and Discussion

### 2.1. Syntheses and Characterization

Our previous studies have indicated the best activity was observed for the chloride Ru(IV) complex in which the protonated ligand acted as a counter ion [16]. Thus, in this paper, complexes **1** and **2** were formed by reacting mother solution ([RuCl_6_]^2−^/[RuCl_6_]^3−^) [20] with the N,O-donor ligands (L1 and L^1^2) in the presence of an EtOH/CH_3_CN/HCl mixture.

**Scheme 1:**
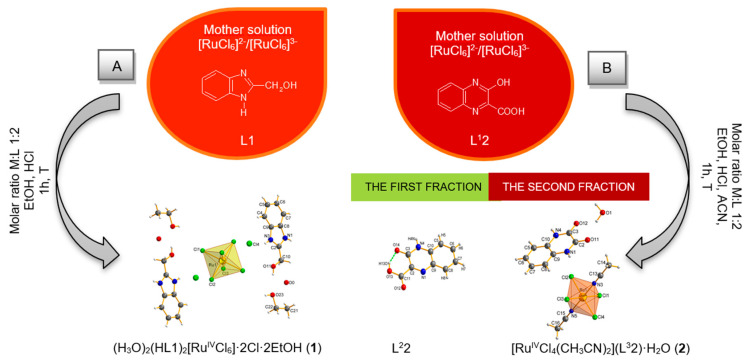
Synthesis of the ruthenium complexes **1** (**A**) and **2** (**B**).

The L1 molecules present in the solution are protonated (in the presence of HCl), and as a result, one of the coordination sites in the N,O-donor ligand is blocked. As a consequence, we obtained that hexachloride ruthenate(IV) is balanced by organic counter-ions formed (HL1) (complex **1**, Scheme 1A) and two protonated ligands in comparison to our previous experimental results [20]. Additionally, under the low-temperature conditions of crystallization, we observed the existence of ethanol in the crystal space, which acted as a solvent. The obtained red crystals of complex **1** are stable in air (m. *p*. 190 °C). The characteristic IR absorption frequencies (cm^−1^) of the ligands (L1, HL1) and complex **1** are presented in Table S1. As the protonated form of L1 appears in complex **1**, we obtained its chloride salt (HL1 in the solid state) for comparison. The IR spectrum of HL1 shows a characteristic strong band assignable to ν_(N-H)_ of protonated pyridine-like nitrogen atoms of the heteroaromatic ring [21]. In the IR spectrum of complex **1**, broad absorption bands are assigned to the new peaks of ν_(O-H)_ from the hydronium cations and ethanol molecules.

**Scheme 2:**
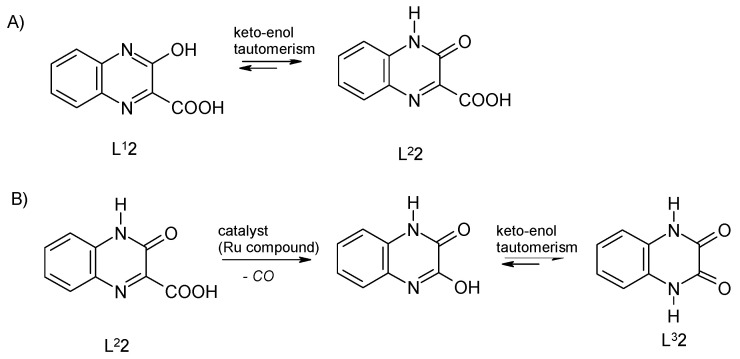
The transformations of ligand L2: (**A**) keto-enol tautomerism of L2 in solvent; (**B**) mechanism of the decarbonylation of L^2^2 by Ru compound.

In the second synthesis, L^2^2 (the first fraction) and complex **2** (the second fraction) (Scheme 1B) were obtained. The commercial ligand (L^1^2) used undergoes the phenomenon of keto-enol tautomerism (Scheme 2A) with the formation of a more stable L^2^2. The presence of bands in IR spectrum of L^2^2 corresponding to ν_N-H_, ν_C=O (COOH)_, ν_C=O_ and ν_C-O_ confirms the tautomer L^2^2 existence in crystal form (Table S1). The resulting complex **2** consists of a neutral Ru(IV) complex with chloride ions and coordinating acetonitrile molecules and a new ligand formed in situ. According to Scheme 2B, most likely due to the presence of Ru(IV) compound, L^2^2 is transformed to 1,4-dihydroquinoxaline-2,3-dione (L^3^2). This process is decarbonylation and it takes place in the -COOH group [22,23]. The decarbonylation is associated with the loss of a CO molecule and forming L^3^2. The mechanism of decarbonylation with enol-form intermediate is presented in Scheme 2B. The absence of a strong IR band associated with ν_C=O_ (-COOH) in the spectrum of complex **2** clearly indicates a lack of carboxylic groups. However, new bands are attributed to ν_C≡N_ vibrations from acetonitrile.

### 2.2. Description of the Molecular Structures

**Figure 1:**
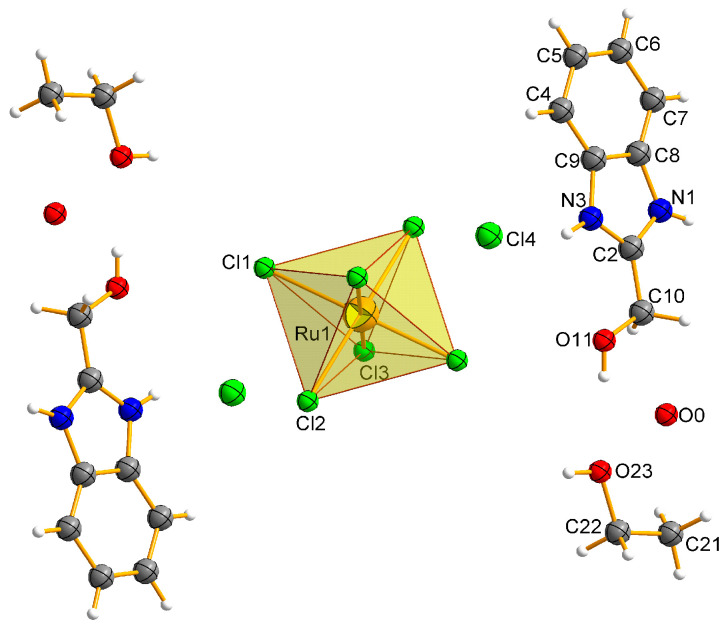
A view part of the (H_3_O)_2_(HL1)_2_[Ru^IV^Cl_6_]·2Cl·2EtOH structure corresponding to the unit formula and coordination polyhedron of complex **1**.

A single-crystal X-ray study revealed that the asymmetric part of the unit cell of complex **1** consists of ruthenium(IV) metal ions located on a symmetry inversion centre (site occupation 0.5) and three coordinating chloride anions (Ru-Cl(1) 2.3719(8), Ru-Cl(2) 2.3817(9) and Ru-Cl(3) 2.3674(8)) in general positions, one protonated 2-(hydroxymethyl)-1H-benzimidazol-3-ium ion (HL1), one chloride counter-anion (Cl^−^), one hydronium cation, and one ethanol molecule. The structure of complex **1** is best represented by the formula (H_3_O)_2_(HL1)_2_[Ru^IV^Cl_6_]·2Cl·2EtOH and is shown in Figure 1, together with the adopted atom numbering scheme. The geometry around Ru(IV) resembles an octahedral geometry, and the six Cl^−^ ions are placed at a rather long but almost symmetric distance of approximately 2.37 Å. Selected bond distances and bond angles are listed in Table S2.

**Figure 2:**
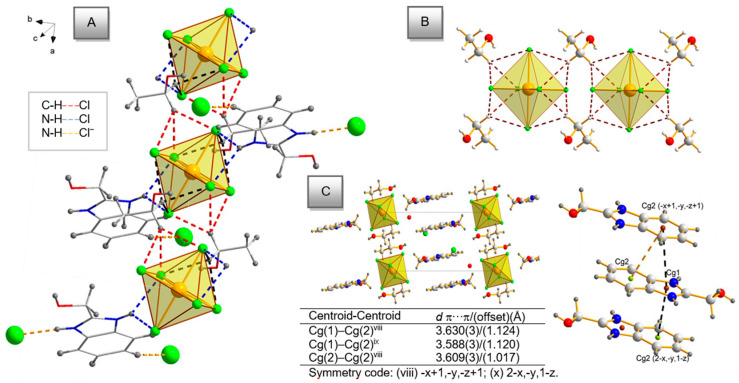
Crystal packing of complex **1** with supramolecular architecture: (**A**) a view of the coordination environment of Ru(IV) ion containing N-H···Cl and C-H···Cl hydrogen bonding; (**B**) the network of hydrogen-bonded ribbons along [1 0 0] of alternating hexachlororuthenium(IV) and ethanol units (ethanol molecules act as ”molecular clips”); (**C**) the layers parallel to (0 1 0) of discrete centrosymmetric [RuCl_6_]^2−^ octahedra and 2-(hydroxymethyl)-1*H*-benzimidazol-3-ium ions and the geometry of the π···π stacking interactions (Cg(1): 5-membered ring defined by: N(1), C(2), N(3), C(9), C(8) atoms; Cg(2): 6-membered ring defined by C(4), C(5), C(6), C(7), C(8), and C(9) atoms).

The crystal structure of complex **1** consists of alternating layers parallel to (010) of discrete centrosymmetric [Ru^IV^Cl_6_]^2−^ octahedra and 2-(hydroxymethyl)-1H-benzimidazol-3-ium ions (Figure 2A). Analysis of the crystal structure at the supramolecular level reveals that the complex molecules are held together by hydrogen bonds of the N-H···Cl and C-H···Cl types and intermolecular π···π stacking interactions between aromatic rings belonging to the 2-(hydroxymethyl)-1H-benzimidazol-3-ium ion (Table S3, and Figure 2A,C). The studies of weak interactions, such as halogen bonds, have received attention only in more recent years. The X-H···Cl (X = N and C) hydrogen bonds have been well appreciated in crystal engineering because they have shown the capability of playing a crucial role in supramolecular architecture and important feature in functional materials [24]. Nevertheless, the investigation of X-H···Cl hydrogen bonding using inorganic supramolecular synthons is relatively rare. In the structure of complex **1**, one of the most interesting features of the observed H-bonding network is the presence of rare four-centered hydrogen bonds (trifurcated H-bonds) of the C-H···Cl type, which contribute to the structure stabilization (Table S3). One ethanol molecule forms six hydrogen bonds (two trifurcated) via H(22A) and H(22B) hydrogen atoms with six coordinating Ru(IV) chloride anions (Figure 2B) to yield a network that can be described as hydrogen-bonded ribbons (chains) of alternating hexachlororuthenate(IV) and ethanol units along *x*-axis. Therefore, these units act as “molecular clips.” Most of the observed C-H···Cl distances (2.49–2.95 Å, Table S3) are shorter/equal than the sum of the van der Waals radii for the H and Cl atoms (2.95 Å), and can be classified as intermediate contacts (2.52–2.95 Å, where distances ≤ 2.52 Å are termed “short”) [25]. The data appeared to suggest that a variety of C-H···Cl interactions identified in the study play a crucial role in the stabilization of the observed supramolecular assemblies. Single intermolecular C-H···Cl interactions are clearly weak because of the low acidity of the C-H system. A manifestation of the weakness of these interactions is the ease of deformation, resulting in the wide range of C-H···Cl angles observed (Table S3). However, the number of potential donors and the fact that a single chloride counter-anion may play a role in a multi-acceptor system (Figure 2A) may well result in an important general contribution to structure stabilization.

**Figure 3:**
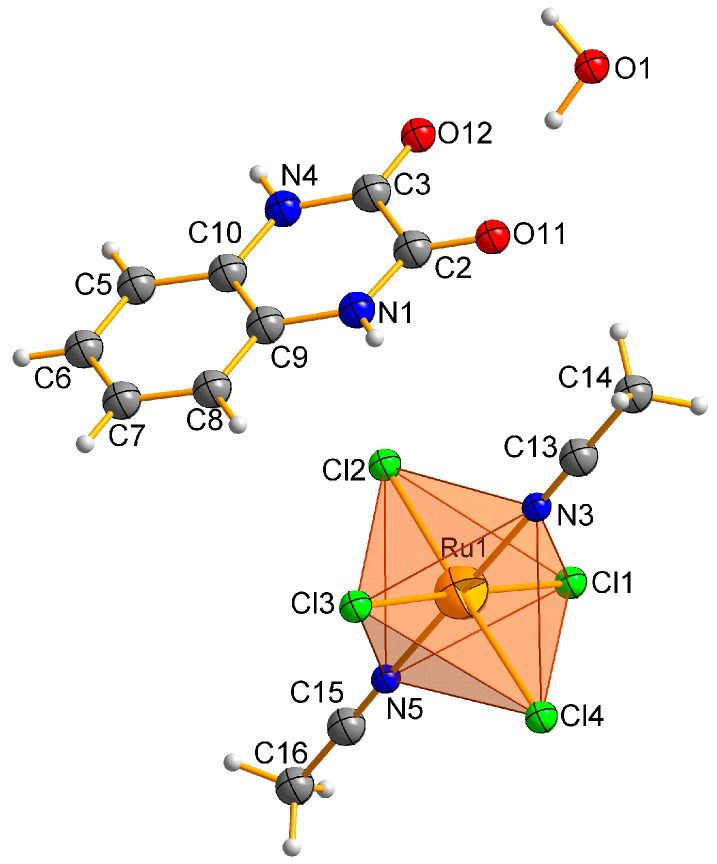
Molecular structure with the atom numbering scheme and coordination polyhedron of complex **2**.

The structure of complex **2** is shown in Figure 3, together with the adopted atom numbering scheme. Ru(IV) is hexacoordinated, with four Clˉ ions placed at rather long but almost symmetric distances (approximately 2.37 Å) and with two N atoms (acetonitrile molecules) 2.0155(2) and 2.0382(2) Å away from it. Thus, the shape of the polyhedron is described as a flattened octahedron. The selected bond lengths and angles are listed in Table S4. The L^3^2 formed in situ belongs to the diketone group of compounds. The bond lengths and angles of L^3^2 are in accordance with the corresponding reported values in the 1,4-dihydro-quinoxaline-2,3-dione core [26,27] and other similar *N*-alkyl quinoxalines [27,28,29]. The existence of L^3^2 in the dione form is evident from the C(2)-O(11) (1.2264(3) Å) and C(3)-O(12) (1.2283(3) Å) bonds, which are shorter than a pure single bond, confirming the double-bond character [30]. The C(2)-C(3) bond is single in nature, compared to multiple-bond character in C(5)-C(6), C(6)-C(7), C(7)-C(8), C(8)-C(9), C(9)-C(10), and C(5)-C(10) (Table S4). The N(1)-C(2) and C(3)-N(4) bonds are significantly shorter than N(1)-C(9) and N(4)-C(10) (Table S4), which are intermediate between those typical for the corresponding single and double bonds, suggesting some degree of delocalization. The N(1)-C(2) and C(3)-N(4) bond lengths are close to the average C*ar*-N*sp*^2^ (planar) value of 1.353(7) Å [30], with the sum of the bond angles around atom N(1)/N(4) (359.97/359.96°) indicating *sp*^2^ hybridization. The heterocyclic ring is approximately coplanar with the benzene ring (torsion angles: C(9)-N(1)-C(2)-C(3), 1.6(4)°, N(4)-C(3)-C(2)-N(1), −1.1(3)°, C(7)-C(8)-C(9)-N(1), 178.8(2)°, and C(2)-N(1)-C(9)-C(10), −1.3(3)°).

**Figure 4:**
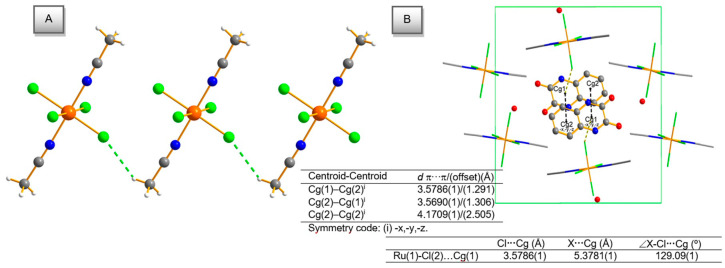
A packing diagram of complex **2**: (**A**) showing the *zig-zag* chains formed by the C-H···Cl hydrogen bonds; (**B**) a view of a channel filled with L^3^2, with marked Ru-Cl···π interactions and π···π stacking interactions (Cg(1): 6-membered ring defined by N(1), C(2), C(3), N(4), C(10), and C(9) atoms; Cg(2): 6-membered ring defined by C(5), C(6), C(7), C(8), C(9), and C(10) atoms).

The crystal packing of complex **2**, shown in Figure 4, is stabilized by the strong or moderate O-H···O and N-H···Cl intermolecular H- bonds, in which the Ru complex is engaged with L^3^2 and water molecules. Interestingly, there is a bifurcated H-bond of the donor type between water and L^3^2. Weak interactions of both C-H···Cl and C-H···O types between L^3^2 and ruthenium complexes contribute to structure stabilization (Table S3). As observed in complex **1**, donor trifurcated interactions are observed the most. There are also some π···π contacts that complete the supramolecular architecture (Figure 4). The centroid distances are in the range of 3.56 Å and correspond well to similar parameters found for a large number of organometallic complexes. The most interesting feature of this H-system is the presence of a Cl···π contact (Figure 4). This type of interaction has played an important role in crystal engineering and has been investigated in much detail only in more recent years. Recent studies clearly demonstrate the potential in the utilization of this weak intermolecular force as an important feature in novel functional materials for potential applications in biochemical processes [31,32,33].

**Figure 5:**
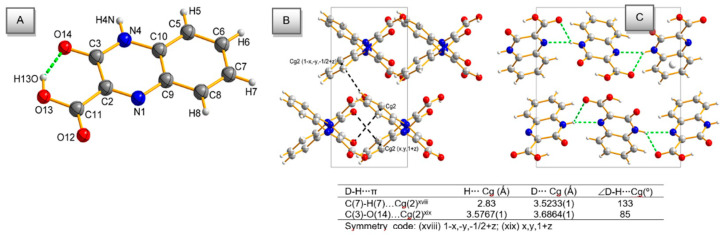
Molecular structure of L^2^2, with the atom numbering scheme and with marked intramolecular H-bond (**A**); (**B**) the infinitive ribbon motive formed by the N-H···N and N-H···O hydrogen bonds; (**C**) the fragment of alternating chains and the geometry of the C-H···π and C-O···π interactions (Cg(2): 6-membered ring defined by C(5), C(6), C(7), C(8), C(9), and C(10) atoms).

The crystal structure of the product isolated as the first fraction (L^2^2) in the preparation of complex **2** is illustrated in Figure 5A. It crystallizes in the orthorhombic space group *P na*2_1_ with four molecules in the unit cell. The present work constitutes the first structural report even though the compound (L^1^2) is available commercially. The existence of L^2^2 in a keto form (Scheme 2) is evident from the C(2)-O(11) bond, which is shorter than a pure single bond. The C(5)-C(6), C(6)-C(7), C(7)-C(8), C(8)-C(9), C(9)-C(10), and C(5)-C(10) bonds have multiple-bond character. The quinoxaline core is essentially planar, with a dihedral angle of 3.09° between the rings of the molecule, which can be attributed to electron delocalization. Present in the molecule is one intramolecular O-H···O hydrogen-bonded ring system formed by carboxylic and carbonyl substituents. The carboxylic group is approximately coplanar with the polycyclic core (torsion angles: N(1)-C(2)-C(11)-O(13), −176.26(2)° and N(1)-C(2)-C(11)-O(12), −176.4(4)°), characterizing typical C-O and C=O bond values of 1.324(3) and 1.198(3) Å, respectively. The selected bond distances and bond angles are listed in Table S5.

In the crystal, L^2^2 molecules are linked into a three-dimensional network via strong intermolecular N-H···O and N-H···N hydrogen bonds (Table S3). The crystal packing is further consolidated by C-H···π and C-O···π interactions between the two symmetry-related face-to-edge quinoxaline cores (Figure 5B).

### 2.3. Molecular Hirshfeld Surfaces

**Figure 6:**
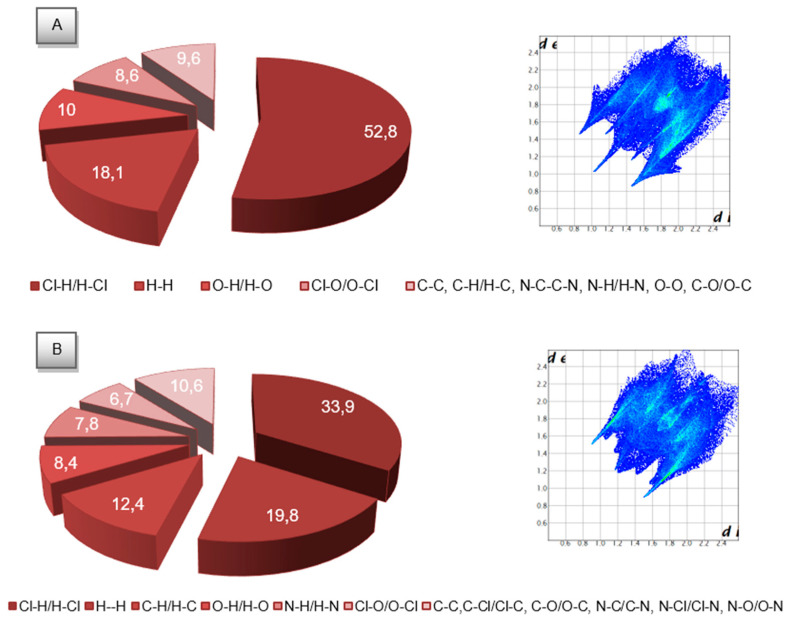
The 2D fingerprint plots of the most significant intermolecular interactions for complexes **1** (**A**) and **2** (**B**) with percentage of interaction.

To obtain additional insight into the role of crystal packing forces in the stabilization of “possible complex-biological target interactions”, we conducted a comparative Hirshfeld surface analysis of complexes **1** and **2**. The molecular Hirshfeld surfaces (*d*_norm_ and *shape index*) of ruthenium(IV) complexes are shown in Figure S1. The 2D fingerprint plots shown in Figure 6 can be deconstructed to highlight particular atom pair contacts. In both chloride complexes, H···Cl/Cl···H and H··· H types of contacts are dominant (Figure S1). A significant difference is observed between the contribution (%) of intermolecular H···Cl/Cl···H types of contacts in the complexes examined, while comparable contributions are found for H-H interactions. As expected, the medium region (complex **1**) is represented by O···H/H···O and O···Cl/Cl···O interactions. In turn, the Hirshfeld surface analysis of complex **2** indicates more types of close contacts in comparison to complex **1**. As dominant interactions, the H···C/C···H contacts (Figure 6B) also make contribution to the Hirshfeld surfaces. Other significant spots in the Hirshfeld surfaces correspond to O···H/H···O, N···H/H···N and O···Cl/Cl···O contacts. Importantly, the analysis of % contribution shows that the polar character of interactions in complex **1** predominates significantly (66.6%), in contrast to that of complex **2**, where these interactions (50.1%) have a percentage similar to that of interactions possessing a lower difference of electronegativity between pair contacts. This difference probably contributes to the slightly higher anti-biofilm activity of complex **1**.

### 2.4. Spectroscopic, Magnetic and Solution Conductivity Properties

#### 2.4.1. UV-Vis Spectroscopy in Solid-State and Aqueous Solutions

The electronic spectra in solid-state (the diffuse reflectance spectrum) and aqueous solutions of HL1 and complex **1** are shown in Figure S2. Analysis of the UV-Vis spectra of complex **1** indicated that strong, moderate intensity or weak bands are related to the intra-ligand π→π*, the ligand–to–metal charge transfer (LMCT, πCl→t_2g_Ru) and the d–d transitions (Table S6). The last band in the visible part of the spectra with a maximum at ~547 nm (the diffuse reflectance spectrum) and ~590 nm (the absorption spectrum) is attributed to the *d–d* transition. According to the Tanabe–Sugano diagram, the *d–d* transition has been assigned to the ^3^T_1g_→^3^E_g_ transition for low-spin Ru(IV) complex (t_2g_^4^e_g_^0^ configuration) in an O_h_ environment. The data obtained for the reflectance spectrum of complex **1** are in agreement with the experimental data in aqueous solution.

More complicated electronic spectra were recorded for the ligand L^2^2 and complex **2** (Figure S3). It is noteworthy that, in the process of complexation, the ligand was transformed into a diketone, and the bands corresponding to the *n*→π* transitions are in the 280–350 nm region in the spectrum of compound **2**. The next bands in the electronic spectrum of the complex were assigned to CT transitions (π(L)→d(Ru)) (Table S6).

#### 2.4.2. Luminescent Properties

The emission spectra for the two ruthenium complexes in an aqueous solution at room temperature are presented in Figure S4. Both complexes show blue luminescence in H_2_O solution (concentration: 2 × 10^−3^ M) at room temperature, with λ_max_ at 351 nm (complex **2**) and 363 nm (complex **1**) upon excitation at 235 and 273 nm, respectively. The quantum yields (Φ) of the compounds were determined to be 0.017 and 0.022, respectively.

#### 2.4.3. Magnetic Measurements

Magnetic susceptibility measurements at room temperature indicated the paramagnetic nature of the studied ruthenium complexes. The effective magnetic moment values of 2.46 μ_B_ for complex **1** and 2.34 μ_B_ for complex **2** are a bit smaller than the spin-only value (2.83 μ_B_). The obtained μ_eff_ values correspond to two unpaired electrons suggesting a low spin t_2g_^4^e_g_^0^ configuration for the Ru(IV) ions in an octahedral environment.

#### 2.4.4. Solution Conductivity

The molar conductivity values for Ru complexes were measured in ethanol at room temperature (298 K). For compound **2**, measured calculations leads to a value of 11 Ω^−1^⋅cm^2^⋅mole^−1^. The value of Λ_M_ exists in the range of non-electrolytes for compound **2** [34]. Attention should be drawn to the compound **1**, for which the value of Λ_M_ is 54 Ω^−1^⋅cm^2^⋅mole^−1^ and it exceeds range of 1:1 electrolytes [34]. The proposed ratio of 3:4 (1:1.33) seems to be rational.

### 2.5. Electrochemical Studies

**Figure 7:**
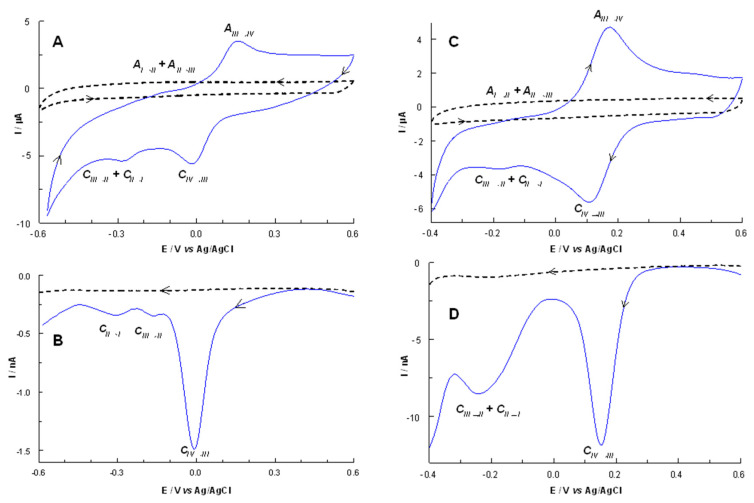
CV (**A**,**C**) and DPV (**B**,**D**) curves recorded in acetonitrile/ethanol solution containing 0.1 M TBAPF_6_ and 1 mM ruthenium complexes **1** and **2** (―) or 1 mM ligands (^_ _ _^) on the first day of the investigations, (CV conditions: GCE, Ø = 2 mm, scan rate 0.1 V s^−1^, T = 25 °C; DPV conditions: CF, Ø = 33 μm, pulse amplitude 20 mV, pulse width 80 ms, scan rate 0.02 V s^−1^). The potential scan started at 0.6 V in negative direction.

The curves recorded with the use of CV and DPV techniques on a glassy carbon (GCE) and carbon fiber (CF) electrodes, respectively, are presented in Figure 7. The voltammetric data obtained from these experiments are given in Table S7.

**Figure 8:**
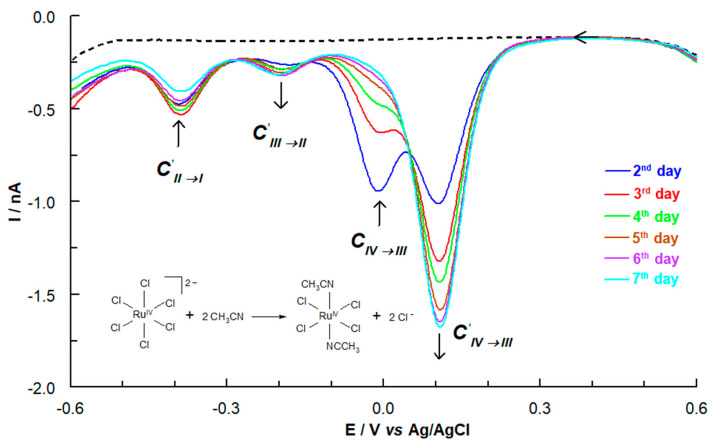
Periodically recorded DPV curves for complex **1**. The presented curves were performed up to 7 days from the preparation of the solution. Conditions as shown in Figure 7.

Based on the CV voltammograms of the ruthenium complexes (Figure 7A,C), it was found that the first redox pairs are attributed to the reduction of Ru(IV) to Ru(III) and the oxidation of Ru(III) to Ru(IV) (*E*_pc_ = −0.004 V, *E*_pa_ = 0.128 V for complex **1**; *E*_pc_ = 0.109 V, *E*_pa_ = 0.169 V for complex **2**, scan rate of 0.1 V·s^−1^). The broad signals observed at potentials of approximately −0.31 V and −0.16 V (complex **1**), and −0.18 V and −0.13 V (complex **2**), can be attributed to the overlapping of the signals connected with the reduction of Ru(III) to Ru(II) and Ru(II) to Ru(I) and the oxidation of Ru(I) to Ru(II) and Ru(II) to Ru(III), respectively. The use of the DPV technique allowed us to separate adjoining peaks. In Figure 7B, two separated signals are observed at −0.16 V for the C_III__→II_ peak and at −0.32 V for the C_II__→I_ peak. In turn, in Figure 7D, the wide signal corresponding to the sum of the peaks C_III__→II_ and C_II__→I_ is recorded at potential of approximately −0.25 V. To verify the reversibility of the redox couples and the number of electrons exchanged, CV diagnostic criteria were considered. The value of the peak-to-peak separation (Δ*E*_p_ for V = 0.1 V·s^−1^) for the redox pair Ru(IV)/Ru(III) in complex **1** significantly exceeds the theoretical value of 0.058 V for a reversible one-electron redox couple [35]. This result gives evidence indicating the irreversible nature of the Ru(IV)/Ru(III) system. In turn, in complex **2**, the value of Δ*E*_p_ = 0.060 V for the Ru(IV)/Ru(III) redox pair is similar to the theoretically expected value, which indicates a reversible process. The reversibility of the electrode process was also examined using the peak width at the half-height of the curves recorded using the DPV technique. The W_1/2_ value obtained for the Ru(IV)/Ru(III) redox peak in complex **1** exceeds the theoretical parameter (W_1/2_ = 0.0904/*n* V) [35], while in complex **2**, it is comparable (Table S7). These data confirm the conclusions obtained from CV experiments. The nature of Ru(III)/Ru(II) and Ru(II)/Ru(I) reversibility is difficult to determine using the CV diagnostic criteria. The DPV criteria applied for Ru(III)/Ru(II) and Ru(II)/Ru(I) signals in complex **1** indicate their irreversible nature. Determination of the W_1/2_ parameter for the same signals in complex **2** proved to be infeasible. The diagnostic criteria characteristic of cyclic and differential pulse voltammetry confirm that all of these redox pairs are associated with the exchange of one electron. Additionally, electrochemical studies of the organic ligand have shown that in the selected potential range (0.6 to −0.6 V), it is not electrochemically active (dashed lines in Figure 7, Figure 8 and Figure S5).

The presented CV and DPV investigations have shown that interesting conclusions may be drawn for the studied system (complex **1**), taking the effects of acetonitrile on the change in the ruthenium complex composition. To the best of our knowledge, our paper is the first in the literature to investigate the mechanism of ligand exchange using DPV method. We noticed that the RuCl_6_^2-^ anion in complex **1** is involved in the exchange of two chloride anions for two acetonitrile molecules (Figure 8) giving composition of compound similar to complex **2**. This effect can be well illustrated by a series of DPV curves recorded every day for seven days (Figure 8).

The curve recorded on the first day as presented and interpreted in Figure 7B, exhibited three cathodic peaks (C_IV__→III_, C_III__→II_ and C_II__→I_). The series of DPV curves obtained during the following six days (from days 2 to 7; Figure 8) exhibited the following results:

∙ The DPV curve recorded on second day shows four signals: C_IV__→III_, C^’^_IV__→III_, C^’^_III__→II_ and C^’^_II__→I_. The cathodic C^’^_IV__→III_ peak appeared as a new peak (0.11 V) and this was connected with formation of [RuCl_4_(CH_3_CN)_2_].
∙ The C_IV__→III_ peak at −0.01 V corresponding to the existence of the RuCl_6_^2-^ complex gradually disappeared over time. Consequently, this disappearance led to an increase in the intensity of the C^’^_IV__→III_ peak (0.11 V), which in turn is characteristic of the [RuCl_4_(CH_3_CN)_2_] complex. This suggests that +IV oxidation state for ruthenium ions is more stable in the acetonitrile coordination sphere than in the chloride coordination environment.
∙ It is worth noting that the C_IV__→III_ signal (0.15 V) in complex **2** is observed at a very similar potential value.
∙ The C^’^_III__→II_ and C^’^_II__→I_ peaks in comparison to the C_III__→II_ and C_II__→I_ peaks are shifted to more negative potentials (Table S7). Additionally, the C^’^_II__→I_ peak current was decreased in favor of an increase in the C^’^_III__→II_ peak.
∙ The exchange of chloride ions for acetonitrile molecules resulted in a decrease in the electron density at the metal centre and made it easier reduce Ru(IV) ion.

**Figure 9:**
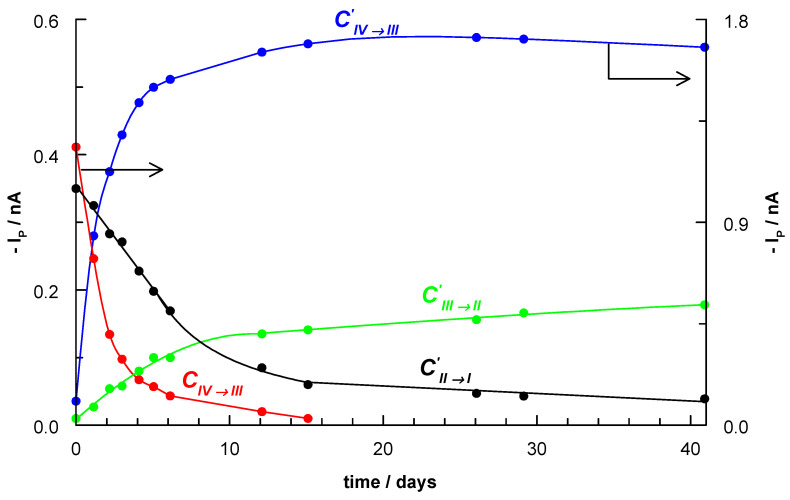
The changes of DPV peak currents for complex **1** over time.

When the equilibrium state was achieved, the total disappearance of the C_IV__→III_ peak was observed, as well as the complete domination of the signal at 0.11 V attributed to C^’^_IV__→III_ (for the [RuCl_4_(CH_3_CN)_2_] complex) (Figure S5). The CV data confirm that the reversibility of electrode processes for three redox couples are changed (Table S7). The confirmation of the described changes illustrates the dependence of the peak currents (DPV curves) on the measurement time presented in Figure 9. In the case of C^’^_IV__→III_ a tendency for a growth of the signal is observed, while the peak currents for C_IV__→III_ are decreased successively. A similar upward trend but with a smaller degree of curve slope is observed for C^’^_III__→II_.

### 2.6. Biological Studies of Ruthenium(IV) Complexes

#### 2.6.1. Anti-Biofilm and Antibacterial Activity

The chloride ruthenium(IV) complexes, the ligands, and metal salt RuCl_3_ were tested for their anti-biofilm activity against *P. aeruginosa* PAO1 and *P. aeruginosa* LES B58. The use of the *P. aeruginosa* PAO1 (reference strain) and *P. aeruginosa* LES B58 (clinical isolate strain) in our research is justified by their virulence in chronic lung infection, antibiotic multi-resistance and strong biofilm production abilities [36,37]. The effects of the compounds on biofilm formation by *P. aeruginosa* strains were analysed in terms of total biomass, and morphology by epifluorescence microscopy. Biofilm biomass was assessed spectrophotometrically by reading crystal violet absorbance. To determine the antibacterial action of complexes **1** and **2** on planktonic cells of bacteria, the broth microdilution method was used, with streptomycin as the reference antibiotic. The prepared compounds were tested against *S. aureus*, *E. coli*, *P. aeruginosa* PAO1 and *P. aeruginosa* LES B58 strains.

**Figure 10:**
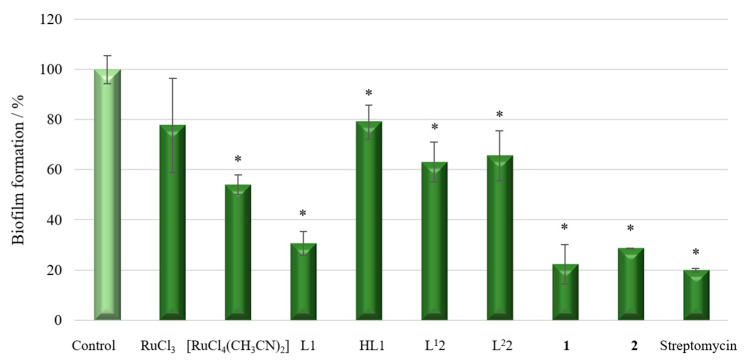
*P. aeruginosa* PAO1 biofilm formation in the presence of RuCl_3_·xH_2_O, [RuCl_4_(CH_3_CN)_2_]·2H_2_O, free and protonated ligands, and ruthenium complexes (concentrations of compounds—1 mM). The absorbance of the control was considered to represent 100% of biofilm formation (results were considered significant when compared to control; * *p <* 0.05. Data are presented as mean ± SD, *n* = 4).

The results of testing the anti-biofilm activity of the ruthenium complexes, Ru salt and ligands used as substrates are presented in Figure 10. Since the tested systems contain several components, it is important to assess which component of the system contributes to the inhibition of the biofilm significantly. For comparison purposes, the HL1 ligand (protonated form of L1), L^1^2 and [RuCl_4_(CH_3_CN)_2_] were prepared in solid state and their chemical composition was confirmed using the elemental analysis, spectroscopic and X-ray methods. The compounds were also tested for anti-biofilm activity. As showed in Figure 10, the metal salt, and the ligands L^2^2 and L^1^2 did not have significant inhibitory effect on biofilm formation, while L1 and [RuCl_4_(CH_3_CN)_2_] exhibited moderate effectiveness. The results also demonstrated that the ruthenium complexes effectively reduced the biofilm growth of the *P. aeruginosa* PAO1 strain. The biofilm formation was inhibited by 78% (complex 1) and 71% (complex **2**) when the concentration of complexes was 1 mM. It is worth noting that these values are similar to those obtained for streptomycin (the reference standard). The result of anti-biofilm test for complex **1** show higher activity than for HL1. Interestingly, L1 indicates better anti-biofilm activity than protonated form of L1. The 2-hydroxymethylbenzimadazolium was able to inhibit biofilm formation, reducing the biomass by 21%, only. Therefore, it is clear that the effect of the RuCl_6_^2−^ anion in a structure 1 on biological activity is significant. The results show that complex **2** is more active than [RuCl_4_(CH_3_CN)_2_] (see Figure 10). It is probable that ligand prepared in situ only slightly enhances the anti-biofilm activity of [RuCl_4_(CH_3_CN)_2_]. *P. aeruginosa* LES B58 biofilm was formed in small quantities under the conditions of the experiment and therefore the results were not subjected to further analysis and interpretation.

Other inhibitory effect of the investigated compounds observed on the planktonic growth of bacteria. The test results (Table S8) showed that only complex **2** exhibits bacteriostatic activity and inhibits the growth of all bacteria investigated (MIC value 1 mM, 813 μg/mL). The rest of compounds were found to be slightly active (complex **2**) or inactive against the strains (ruthenium salt and ligands) at the concentrations tested.

**Figure 11:**
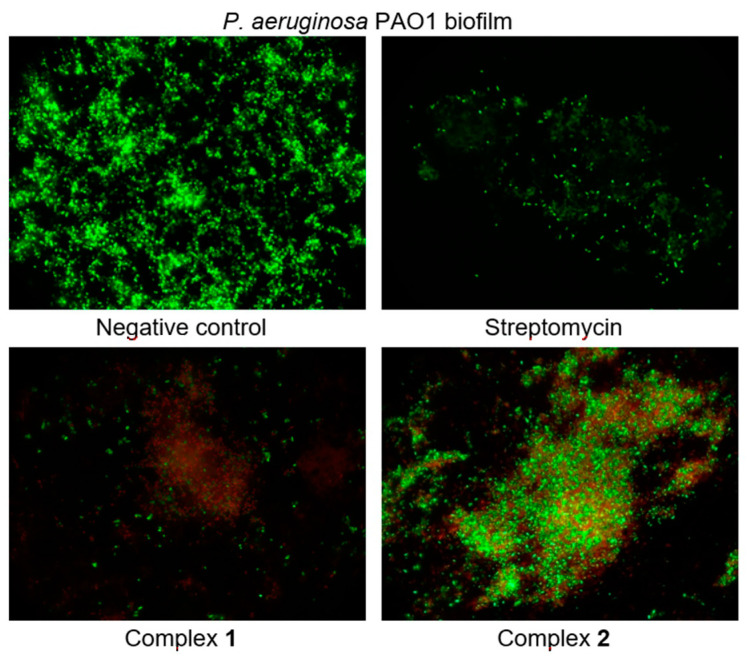
Epifluorescence microscopy images of *P. aeruginosa* PAO1 biofilm treated with 1 mM of ruthenium complexes. Biofilm was stained with nucleic acid stains using the FilmTracer™ LIVE/DEAD Biofilm Viability Kit (Invitrogen, Carlsbad, CA, USA; live cells are represented by the colour green; dead cells are represented by the colour red). The epifluorescence microscopy images were captured at 1000× magnification.

We have also evaluated the viability and survival ability of bacteria in the biofilm layers based on a fluorescence Live/Dead assay by exposure of *P. aeruginosa* PAO1 to complexes at 1 mM concentrations. The BacLight™ Live/Dead Bacterial Viability Kit (Invitrogen, Carlsbad, CA, USA) used consists of two dyes, SYTO-9 and propidium iodide (PI), which both stain nucleic acids. Green fluorescing SYTO-9 is able to enter all cells and is used for assessing total cell counts (active bacterial cells), whereas red fluorescing PI enters only cells with damaged cytoplasmic membranes (dead bacterial cells). Figure 11 shows the effect of the ruthenium complexes on *P. aeruginosa* PAO1 biofilm architecture. As can be seen on representative images (Figure 11), the structure of the *P. aeruginosa* PAO1 biofilm has changed under the influence of ruthenium complexes. The presence of compounds in the culture caused the aggregation of cells into microcolonies, which was not observed in the control. Moreover, the ruthenium complexes resulted in the death of many bacterial cells. The ratio of dead to live cells was significantly higher in the case of complex **1**-treated cells as compared with the complex **2**-treated cells. This result correlates well with the data obtained by crystal violet staining.

The activity of the chloride ruthenium complexes against the *P. aeruginosa* PAO1 biofilm, described in this paper, is higher than the activity of the chelate ruthenium complexes with N-heteroaromatic carboxylic acids and slightly lower than the activity of the benzimidazole-based ruthenium(IV) complex (these results have been published by our group most recently) [16,17]. Our results show that the ruthenium complexes have a high potential to inhibit biofilm formation. The observed anti-biofilm effect apparently refers to inhibiting adhesion of the bacterial strain to microtiter plate surface. The ability to form hydrogen bonds with the various components of the matrix (like extracellular polymeric substances) probably interferes with cell metabolism, triggering cell death. It is speculated that the chloride ruthenium complexes may lead to easier penetration into the bacterial cell for an anti-biofilm effect against *P. aeruginosa*. The chloride ruthenium complexes were also found to be more active against the *P. aeruginosa* PAO1 biofilm than against the planktonic form. This observation is clinically important, as bacteria living in a biofilm can be up to 1000 times more resistant to antimicrobial agents than planktonic bacteria.

#### 2.6.2. Estimation of Oxidative Damage Based on Digestion of Plasmid DNA with Fpg Protein

**Figure 12:**
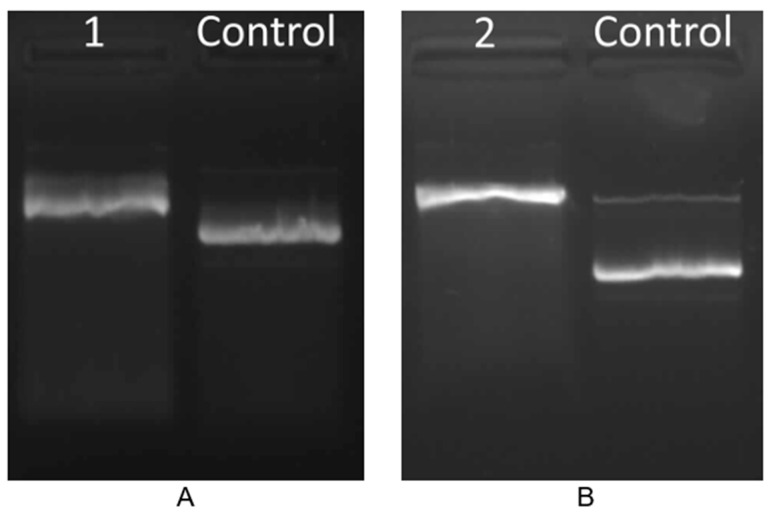
Estimation of DNA damage induced by complex **1** (**A**) and complex **2** (**B**) based on pUC19 plasmid DNA isolated from *E. coli* DH5α and digested by Fpg protein.

To test the ability of the chloride ruthenium complexes to interact with intracellular DNA, mainly due to generation of oxidative stress in bacterial cells, formamidopyrimidine-DNA glycosylase (Fpg) was used to digest modified purines in DNA. *E. coli* was treated with the chloride ruthenium complexes and plasmid pUC18 DNA was isolated. Samples treated with complexes **1** and **2** were not digested (control) as shown in Figure 12, where changes in the topological forms of plasmids and smearing were evident after the Fpg digestion. In the case of both complexes, the *ccc* form was dominant in control, while after Fpg digestion the *ccc* form was linearized. However, the results differed between complexes, where in case of complex **2** assay indicated more extensive damage of plasmid DNA than for complex **1**.

The use of Fpg protein known as 8-oxoguanine DNA glycosylase (or formamidopyrimidine [fapy]-DNA glycosylase) is significant since it removes from double stranded DNA a broad spectrum of oxidized and alkylated bases: 7, 8-dihydro-8-oxoguanine, 8-oxoadenine, unsubstituted and substituted imidazole ring-opened purines introduced into DNA by hydroxyl radicals (e.g., FapyG, FapyA), as well as by chemical carcinogens, including anticancer drugs (e.g., Fapy-7MeG, Fapy-7EtG, Fapy-7aminoethylG, aflatoxin B_1_-fapy-guanine, 5-hydroxy-cytosine, and 5-hydroxy-uracil) [38,39,40,41]. Two additional activities characterize the Fpg protein: (i) the AP-lyase activity which cleaves both 3’ and 5´ to the AP site thereby removing the AP site and leaving a 1 base gap by β-δ-elimination, and (ii) a dRPase activity which removes the 5´ terminal deoxyribose phosphate from DNA incised by an AP endonuclease [42,43].

### 2.7. Regularity between Electrochemical Properties and Biological Activity

It emphasizes significance of the electrochemical techniques in analysis of synthetic products, in their relationship with bioactive properties, mainly antimicrobial, antifungal, antiparasitical, and antitumor activities. Electrochemical parameters do not give absolute correlation with biological activity data, due to the enormous complexity of the biomedical chemistry [44]. However, it can indicate some regularities between the factors. It is worth noting that examples where electrochemistry, dealing with different aspects of electron transfer (ET), contributes significantly to biomedical chemistry [44]. Many of the most important physiological processes are based on redox chains involving numerous successive enzyme-catalyzed processes. There is a set of similarities between electrochemical and biological reactions concerning electron transfer (ET) pathways [45]. The results of our study indicate that the higher half-wave potential, the lower the biological activity of the ruthenium(IV) complexes. In a previous paper, we have found out the inverse relationship between biological activity and redox potential among chelate complexes [17]. Also, Ruiz-Azuara and colleagues have observed correlation between biological activity and reduction potential [46].

**Figure 13:**
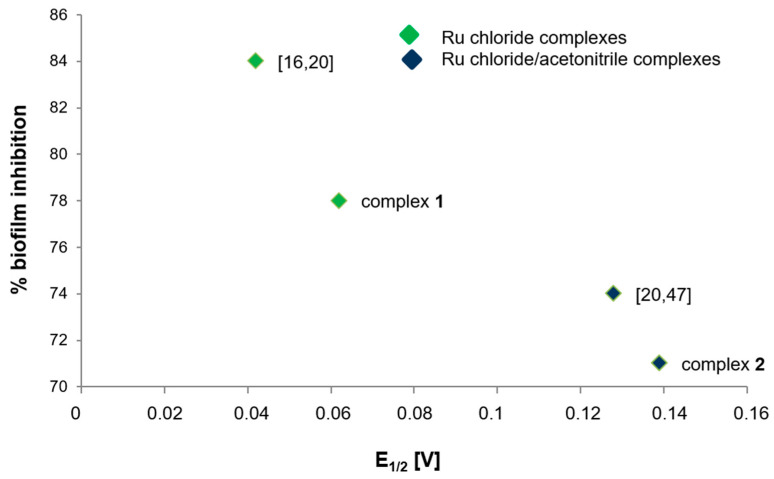
Correlation between the redox potential (*E*_1/2_) for Ru(IV)/Ru(III) couple and the percentage biofilm inhibition against *P. aeruginosa* PAO1 strain [16,20,47].

The correlation tendency between the biological activity and the electrochemical evaluations of chloride ruthenium(IV) compounds (studied by our team so far) was carried out by CV/DPV yields interesting results. The electrochemical behaviour was similar in Ru(IV)/Ru(III) cases studied (*E*_1/2_ 0.1–0.15 V) [17,20]. The correlation of the biological activity with half-wave potentials was made on the Ru(IV)/Ru(III) couple, reported as the stable formation of complexes. The correlation of *E*_1/2_ with % biofilm inhibition had a decreasing tendency according to Figure 13, the higher half-wave potential, the lower the biological activity. Moreover, we have found out that chloride Ru(IV) complexes indicated higher biological activity than chloride/acetonitrile complexes.

## 3. Materials and Methods

Details of the materials, syntheses of compounds, equipment and experimental procedures used were presented in the Supporting information. The most important structural parameters of the **1**, **2** and L^2^2 were given in Table S9 (electronic supplementary material).

## 4. Conclusions

In summary, as a result of the synthesis, we have obtained new chloride ruthenium(IV) complexes, and their spectral, structural, and electrochemical characterization has been performed. An additional advantage of the preparation of complex **2** was that the L^2^2 ligand was obtained as crystals and that its structure was solved, which has not been reported in the literature so far. In the obtained complexes, metal in +IV oxidation state possesses an octahedral environment. The intermolecular classical and rare weak hydrogen bonds, and Clˉ···π, π···π contacts significantly contribute to structure stabilization and played a decisive role in the supramolecular architecture and application of the presented complexes as functional materials. Importantly, the Hirshfeld surface analysis shows that predominance of the polar character of interactions in relation to the interactions possessing a lower difference of electronegativity between pair contacts probably contributes to the higher anti-biofilm activity of complexes. The electrochemical studies confirmed that the presence of acetonitrile molecules in complex **2** resulted in a decrease in the electron density at the metal centre and made it easier reduce Ru(IV) ion. Biological studies of the tested Ru(IV) complexes have confirmed their anti-biofilm activity against *P.* *aeruginosa* PAO1, where complex **1** has exhibited slightly higher activity than compound **2**. Moreover, the activity against the biofilm was found to be definitely greater than that against the planktonic bacteria, which makes the complexes selective. The correlation of *E*_1/2_ with % biofilm inhibition had a decreasing tendency, the higher half-wave potential, the lower biological activity. Based on the results obtained, it can be stated that the high-valent Ru complexes can generate a cell response similar to oxidative stress in bacterial cells. Moreover, the Ru-complexes probably induce oxidative DNA damage in living cells and this effect can be shown in bacteria lacking a repair system. The use of the presented ruthenium complexes paired with antibiotics in the treatment of infection might be an effective method for bacterial eradication. Such interactions will be explored in our future research. Taking into account the strong resistance of *P. aeruginosa* PAO1 to various agents, these results may provide information for the development of important antibacterial solutions in the future.

## Supplementary Materials

The following are available online, Figure S1: The Hirshfeld surfaces of complexes **1** (A) and **2** (C) mapped with 3D *d*_norm_ (with transparency enabled) and shape index functions (B and C), Figure S2: UV-Vis absorption spectra of solid state (A) and in aqueous solution (B) for complex **1** and HL1, Figure S3: UV-Vis absorption spectra in aqueous solution for complex **2** and L^2^2, Figure S4: Emission spectra of ruthenium complexes in an aqueous solution, Figure S5: CV (A) and DPV (B) voltammograms of 1 mM complex **1** recorded on the last day of the investigations. Conditions as shown in Figure 7, Table S1: The characteristic IR absorption frequencies (cm^−1^) of the ligands (L1, HL1, L^2^2) and the ruthenium complexes, Table S2: Selected bond lengths (Å) and valence angles (°) for (H_3_O)_2_(HL1)_2_[Ru^IV^Cl_6_]·2Cl·2EtOH, Table S3: Hydrogen bonds for the ruthenium complexes and L^2^2 (Å) and (°), Table S4: Selected bond lengths (Å) and valence angles (°) for [Ru^IV^Cl_4_(CH_3_CN)_2_](L^3^2)·H_2_O, Table S5: Selected bond lengths (Å) and valence angles (°) for L^2^2, Table S6: UV-Vis spectroscopic data for HL1, L^2^2 and Ru(IV) complexes, Table S7: Electrochemical data (in V vs. Ag/AgCl) for the ruthenium complexes obtained by cyclic voltammetry (CV) on GCE and by differential pulse voltammetry (DPV) on CF disk microelectrode, Table S8: Bacteriostatic activities of the investigated ruthenium complexes, ruthenium salt and ligands as MIC concentrations, expressed in mM and μg/mL, Table S9: Crystal data and structure refinements for **1**, **2** and L^2^2.
