# Supplementary material for: Ruthenium(IV) Complexes as Potential Inhibitors of Bacterial Biofilm Formation
Source: Molecules. 2020 Oct 26;25(21):4938. doi: 10.3390/molecules25214938 (PMC7662803; doi:10.3390/molecules25214938)
Supplement: Supplementary file 1 [file molecules-25-04938-s001.pdf]

Electronic Supplementary Information

Ruthenium(IV) Complexes as Potential Inhibitors of Bacterial Biofilm Formation

Agnieszka Jabłońska – Wawrzycka*[1], Patrycja Rogala[1], Grzegorz Czerwonka[2], Sławomir Michałkiewicz[1], Maciej Hodorowicz[3], Paweł Kowalczyk[4]

[1] *Institute of Chemistry, Jan Kochanowski University in Kielce, 7 Uniwersytecka Str., 25-406 Kielce, Poland,*
e-mail: agajw@yahoo.com; Agnieszka.Jablonska@ujk.edu.pl
[2] *Institute of Biology, Jan Kochanowski University in Kielce, 7 Uniwersytecka Str., 25-406 Kielce, Poland,*
[3] *Faculty of Chemistry, Jagiellonian University, 2 Gronostajowa Str., 30-387 Kraków, Poland*
[4] *Department of Animal Nutrition, The Kielanowski Institute of Animal Physiology and Nutrition, Polish Academy of Sciences, 3 Instytucka Str., 05-110 Jabłonna, Poland*

CONTENTS

1. Experimental section..S2 – S7

2. Supporting tables..S8 – S14

3. Supporting figures..S15 – S18

4. Supporting references..S19 – S20

1. Experimental section

1.1. Materials

$RuCl_3 \cdot xH_2O$, 2-hydroxymethylbenzimadazole, 3-hydroxy-2-quinoxalinecarboxylic acid were purchased from Sigma Aldrich and used as received. The solvents - concentrated hydrochloric acid and acetonitrile were sourced from commercial vendors and used without further purification. Ethanol was acquired from Linegal Chemicals and was purified by using a distillation method. The starting (mother) ruthenium(III) chloride solution (0.1 M) was prepared according to the procedure described in the literature [1].

1.2. Syntheses of ruthenium(IV) complexes

*1.2.1. Synthesis of $(H_3O)_2(HL1)_2[Ru^{IV}Cl_6]\cdot 2Cl\cdot 2EtOH$ (complex **1**)*

A solution of 2-hydroxymethylbenzimidazole (0.1482 g, 1 mmol) in ethanol (5 ml) was added dropwise to a stirring 0.1 M solution of ruthenium(III) chloride (mother solution, 5 ml, 0.5 mmol). Then, ethanol and hydrochloric acid were added cautiously to the mixture (8 : 1 v/v). The reaction solution was heated at reflux for one hour. After cooling to room temperature, the resulting mixture was transferred to a freezer. Passive evaporation of the red-orange solution resulted in red crystals of the complex that were suitable for X-ray investigation. The product was separated by filtration and dried under vacuum. The crystals were collected in 83% yield (337 mg). Melting point: 190°C. Elemental analysis (%), Calc. for $RuCl_8C_{20}H_{36}N_4O_6$: C 29.54, H 4.46, N 6.89; Found: C 29.75, H 4.55, N 7.09. FT-IR (cm^{-1}): 3602(br), 3337(s), 3242(br), 3140(vs), 3043(m), 3031(m), 2965(s), 2897(s), 2825(s), 2729(s), 1655(w), 1626(s), 1564(s), 1487(s), 1456(vs), 1434(vs), 1422(vs), 1148(s), 1122(s), 1113(s), 1082(vs), 1038(m), 692(s), 603(vs).

*1.2.2. Synthesis of $[Ru^{IV}Cl_4(CH_3CN)_2](L^32)\cdot H_2O$ (complex **2**)*

3-Hydroxy-2-quinoxalinecarboxylic acid (L^12(commercial)) (0.1907 g, 1 mmol) dissolved in a mixture of acetonitrile-ethanol (2 : 1 v/v) was added to a 0.1 M solution of ruthenium(III) chloride (5 ml, 0.5 mmol), and the resulting mixture was stirred at room temperature. In the next step of the synthesis, ethanol and concentrated hydrochloric acid were added cautiously to the solution. The reaction mixture was heated at 75°C and stirred for 1 hour; then, it was left to crystallize slowly. After a few days, the first fraction in the form of greenish-gold crystals was isolated and dried in air (L^22). Upon standing at room temperature for 10 days, brownish red crystals (complex **2**) of the second fraction appeared in the orange solution (filtrate). The product was filtered and dried in air. L^22 and complex **2** were isolated in 18% (3.1 mg) and 39% (98.5 mg) yields, respectively. Melting point for

L^22: 267°C; for complex **2**: 277°C. Studies have shown that the L^22 formed is tautomer of 3-hydroxy-2-quinoxalinecarboxylic acid. The tautomeric structure of the obtained product was confirmed by X-ray crystallography and IR experiment. L^22 – Elemental analysis (%), Calc. for $C_9H_6N_2O_3$: C 56.85, H 3.18, N 14.73; Found: C 56.89, H 3.13, N 14.70. FT-IR (cm^{-1}): 3250 - 2090(w, br), 3130(w), 3084(w), 1754(s), 1730(s), 1650(s), 1640(vs), 1624(vs), 1539(m), 1490(s), 1450(vs), 1420(vs), 1150(vs), 1100(vs), 1050(m), 904(s), 820(s), 775(vs), 678(s), 638(s), 595(vs). Complex **2** – Elemental analysis (%), Calc. for $RuCl_4C_{12}H_{14}N_4O_3$: C 28.53, H 2.79, N 11.09; Found: C 28.79, H 3.03, N 11.16. FT-IR (cm^{-1}): 3500(br), 3242(br), 3180(vs), 3050(m), 3040(m), 2996(s), 2932(s), 2294(s), 2250(s), 1700(w), 1680(s), 1655(s), 1625(s), 1564(s), 1510(w), 1480(s), 1442(vs), 1430(vs), 1410(vs), 1389(s) 1370(s), 1148(s), 1030(vs), 1020(m), 962(s), 941(s), 926(s), 764(s), 741(s), 714(s), 632(s), 580(s), 542(vs), 513(s).

1.3. Physical measurements

The elemental analysis (C, H and N) was performed on a Vario Micro Cube Elemental Analyser CHNS. The IR spectra were recorded on a Nicolet 380 FT-IR spectrophotometer in the spectral range 4000 – 500 cm^{-1} using the ATR-diffusive reflection method. UV-Vis spectra of the solid state of ligand – 2-hydroxymethylbenzimidazole and complex **1** were recorded on a Shimadzu 2101 PC scanning spectrophotometer equipped with an ISR-260 attachment. The Kubelka–Munk function ($F(R_\infty)$) [2] was used to convert reflectance measurements into equivalent absorption spectra using the reflectance of $BaSO_4$ as a reference. The multi-peak fitting analysis of the reflectance spectrum (complex **1**) was applied using OriginPro8.5.1 program (OriginLab, Northampton, MA, USA). UV-Vis measurements in aqueous solutions were performed on a V-630 UV-Vis spectrophotometer from Jasco using 1 cm cuvettes against water as reference solutions. The absorbance measurements were recorded ca. 22°C and the concentrations were: $1.13\cdot10^{-4}$ M (for L1), $1.23\cdot10^{-4}$ M (for complex **1**), $9.80\cdot10^{-5}$ M (for L^22) and $9.23\cdot10^{-5}$ M (for complex **2**). The luminescence spectra were measured with an Infinite M200 PRO microplate reader (Tecan) with the xenon flashlamp as a light source (at room temperature). Magnetic measurements were carried out on a magnetic susceptibility balance (Sherwood Scientific) at room temperature by Gouy's method, using $Hg[Co(NCS)_4]$ as a calibrant. The data were corrected for diamagnetic contributions, which were estimated from Pascal's constants [3]. Molar conductivities of freshly prepared $1\cdot10^{-3}$ $mol\cdot dm^{-3}$ EtOH solutions were measured using Jenway. Voltammetric experiments were performed using a Model M161E electrochemical analyser connected with Model M162 preamplifier (mtm-anko, Poland) and controlled *via* a

Pentium computer using mEALab 2.1 software (mtm-anko, Poland). The details of the procedure have been described previously [4]. Electrochemical investigations of ruthenium complexes and free ligands were performed in a mixture of CH_3CN – EtOH (3 : 2, *v/v*) containing 1 mM compound with 0.1 M tetrabutylammonium hexafluorophosphate ($TBAPF_6$) (from Fluka, electrochemical grade) as a supporting electrolyte. The electrochemical properties of complexes **1** and **2** were studied by cyclic voltammetry (CV) on glassy carbon electrode (GCE) (2 mm in diameter A = 0.0314 cm^2 (Mineral, Warsaw)). Some experiments were performed with the use of differential pulse voltammetry (DPV) on carbon fiber (CF) disk microelectrode (33 μm in diameter (BASi, United Kingdom)). DPV voltammograms were registered using a pulse amplitude of 20 mV, pulse width of 80 ms and scan rate of 20 mV s^{-1}. This technique is considered a convenient method because of its good sensitivity selectivity and resolution of the signals, limited influence of adsorption phenomena on recorded curves and thus excellent reproducibility [5].

1.4. Crystal structure determination and refinement (XRD)

Diffraction intensity data for single crystal of complex **1** was collected at room temperature on a KappaCCD (Nonius) diffractometer with graphite-monochromated MoK_α radiation (λ = 0.71073 A). Corrections for Lorentz, polarization and absorption effects [6,7] were applied. The structure was solved by direct methods using the program package SIR-92 [8] and refined using a full-matrix least square procedure on F^2 using SHELXL-2016/6 [9,10]. Anisotropic displacement parameters for all non-hydrogen atoms and isotropic temperature factors for hydrogen atoms were introduced. In the structure the hydrogen atoms connected to carbon atoms were included in calculated positions from the geometry of molecules. In the crystal lattice of **1**, the presence of two hydronium cations was observed. There is no indications in the difference density map as to the location of the H atoms belong to O(0).

Diffraction intensity data for single crystal of complex **2** and $L^2$2 were collected at 120 K on the Oxford Diffraction Super Nova diffractometer using monochromatic Mo Kα radiation, λ = 0.71073 Å. Cell refinement and data reduction were performed using firmware.[11] Positions of all of non-hydrogen atoms were determined by direct methods using SHELXL-2016/6 [9,10]. All non-hydrogen atoms were refined anisotropically using weighted full-matrix least-squares on F^2. Refinement and further calculations were carried out using SHELXL-2016/6 [9,10]. All hydrogen atoms joined to carbon atoms were positioned with an idealized geometries and refined using a riding model with U_{iso}(H) fixed at 1.2 U_{eq} (C_{arom}). The positions of all hydrogen atoms were constrained for all compounds

using AFIX (SHELXL) commands. The positions of hydrogen atoms of water molecules (complex **2**) have been refined with restrains for ideal water molecules. The final structure models have been refined with constrains for positions of first time refined water hydrogen atoms with restrains. The crystallographic data and detailed information on the structure solution and refinement for the ruthenium complexes (**1** and **2**) and $L^2$2 are given in Table S9. The figures were made using DIAMOND [12] software. CCDC 1059868, 1996910 and 1996909 contain the supplementary crystallographic data for **1**, **2** and **$L^2$2**, respectively. These data can be obtained free of charge from The Cambridge Crystallographic Data Centre *via* www.ccdc.cam.ac.uk/data_request/cif.

The XRD investigation was carried out on a DRON-2 (Russia) diffractometer connected to an IBM computer, stepwise, over the 2θ angle range 10-75°, using $CuK_α$ radiation. The products of decomposition were studied using X-ray powder method identified on the basis of ICDD using XRAYAN package [13].

1.5. Hirshfeld surface analysis

Molecular Hirshfeld surfaces calculations were performed using the Crystal Explorer package *ver.* 3.1 [14]. When the .cif file of the title compounds was entered into the Crystal Explorer program, all of the bond lengths to hydrogen were automatically modified to the standard neutron values (CH = 1.083 A). Hirshfeld surface analysis included the descriptor d_{norm} and the *shape index* [15]. The calculations and details of analysis were made as described in [4]. The molecular Hirshfeld surfaces of complexes **1** and **2** were generated using a standard (high) surface resolution with the 3D d_{norm} surfaces mapped over a fixed colour scale of −0.372 (for **1**)/−0.313 (for **2**) (red) to 1.385 (for **1**)/1.108 (for **2**) Å (blue). The *shape index* was mapped in the colour range of −1 to 1 (for **1** and **2**). The colour encodes normalized distance to nearest nuclei and thus conveniently illustrates the "strength" of all types of the intermolecular contacts present. In turn, the fingerprint plots provide a quantitative measure of the intermolecular interactions on the surface.

1.6. Biological tests

1.6.1. Bacterial strains and growth conditions

The *in vitro* antimicrobial activity of the ligands and their ruthenium complexes were evaluated against representative Gram-positive (*Staphylococcus aureus* ATCC 6538P) and Gram-negative (*Escherichia coli* ATCC 8739, *Pseudomonas aeruginosa* PAO1 (biofilm model strain) and *Pseudomonas aeruginosa* LES B58 (clinical isolate)) bacteria. The *P. aeruginosa* PAO1 and LES B58 isolates were derived from the International *Pseudomonas aeruginosa* Reference Panel. Panel is available from the Belgian Co-ordinated Collection of Microorganisms (BCCM)/LMG Bacteria Collection, Ghent University, Gent, Belgium (http://bccm.belspo.be/about-us/bccm-lmg).

The bacterial strains studied were cultivated in Trypticase Soy Broth (TSB) medium (Biocorp, Warsaw, Poland) for 18 h at 37°C with shaking (160 rpm). Overnight cultures of bacteria were diluted 1:100 into fresh TSB medium.

Cultures were used as a source of bacteria for minimal inhibitory concentration (MIC) testing as well as biofilm crystal violet staining and LIVE/DEAD fluorescence assays (Filmtracer™ LIVE/DEAD™ Biofilm Viability Kit, Invitrogen, Carlsbad, California, USA).

1.6.2. Minimal inhibitory concentration

A broth microdilution method was used to determine the minimum inhibitory concentrations of the tested samples of the compounds. The ruthenium complexes and the ligands were prepared by dissolving compounds in distilled water. The stock concentrations of tested compounds were 2 mM. The serial two-fold dilutions were made in a concentration range from 1 mM to 0.0625 mM in the sterile 96-well microtiter transparent plates (Greiner, Monroe, NC, USA) containing nutrient broth. After that, diluted suspensions were added to appropriate wells. The inoculated plates were incubated at 37°C for 24 h. The negative control (bacterial culture in the medium) and positive control (antibiotic control – streptomycin) were used as references to determine the growth inhibition of bacteria. The MIC parameter was recorded as the lowest concentration of the compound at which the isolate was completely inhibited (as evidenced by the absence of visible bacterial growth). The experiments were performed using the Infinite M200 PRO microplate reader (Tecan, Männedorf, Switzerland). Tests were conducted as three independent repeats.

1.6.3. Inhibition of biofilm formation

The inhibition effect of the tested compounds on biofilm formation by *P. aeruginosa* PAO1 and LES B58 strains was measured by crystal violet method using 96-well microtiter plates [16]. The amount of biofilm formed was determined as described previously [4]. Stock solutions of test compounds were prepared in distilled water. The final concentrations of compounds in the cell cultures were in the range 0.0625 – 1 mM. Additionally, fresh medium was used as a negative control and streptomycin as a positive control. Absorbance of the eluted crystal violet was measured on an Infinite M200 PRO microplate reader at wavelength of 595 nm (Tecan, Männedorf, Switzerland). Assays were performed at least in three independent experiments.

The measurement results, expressed in absorbance units, were converted into percentages to allow the comparison of numerical data obtained in different experiments.

1.6.4. Live/Dead staining of the bacterial biofilm

Fluorescence microscopy was used to image live/dead cells in the *P. aeruginosa* PAO1 biofilm. First, the *P. aeruginosa* PAO1 biofilm was cultivated in 6-well microtiter plates on glass coverslips

in TSB medium at 37°C for 24 h without shaking. Then, the culture was supplemented with solutions of the ruthenium complexes (concentration: 1 mM). After 24-hour incubation, the coverslips were carefully washed with sterile water in order to remove nonadherent cells. Microcolonies formed on the glass surface were stained with a FilmTracer™ LIVE/DEAD® Biofilm Viability Kit (Invitrogen, Carlsbad, CA, USA) according to the manufacturer's protocol. After 15 minutes incubation at room temperature in the dark, the samples were washed with water to remove the excess dyes. Images were collected with a ZEISS Axio Scope.A1 epifluorescence microscope. The experiments were repeated three times to obtain consistent results.

1.6.5. Statistical analysis

Statistical analysis was performed using one-way analysis of variance (ANOVA). Significance was set at $p<0.05$.

1.6.6. Estimation of oxidative damage based on digestion of plasmid DNA with Fpg protein

The plasmid DNA were isolated from *E. coli* DH5α by using a New England Biolabs Kit (Ipswich, Massachusetts, USA) according to the manufacturer's instructions. The obtained DNA was digested by Fpg protein (New England Biolabs, cat no. M0240S, 8000 U/mL) as follows: Fpg protein was diluted 50-fold with 10× NEB buffer (provided by the Fpg protein manufacturer) and mixed with 100× BSA solution (also supplied with Fpg protein). Next, 8 µL of purified plasmid DNA was mixed with 2 µL of Fpg solution and 2 µL of NEBuffer, and incubated at 37°C for 30 min. Control DNA and digested plasmid DNA (incubated with the tested compounds) samples were evaluated by 1% agarose gel electrophoresis.

2. Supporting tables

Table S1. The characteristic IR absorption frequencies (cm^{-1}) of the ligands (L1, HL1, $L^2$2) and the ruthenium complexes.

**Assignment**	**L1**	**HL1**	**Complex 1**	**$L^2$2**	**Complex 2**
$\nu_{O\text{-}H}$ (H_3O/H_2O; EtOH; L)	– ; –; 3230	–; –; 3240	3602; 3337; 3242	3250 - 2090	3500; – ; 3242
$\nu_{N\text{-}H}$	3099	3274	3140	3130	3180
$\nu_{C\text{-}H\ aromatic}$	3059, 3033	3022, 3013	3043, 3031	3084	3050, 3040
$\nu_{C\text{-}H\ methyl}$	–	–	2965, 2897, 2825	–	2996, 2932
$\nu_{C\text{-}H\ methylene}$	2745	2772	2729	–	–
$\nu_{C\equiv N}$	–	–	–	–	2250, 2294
$\nu_{C=O}$ (COOH, ketone)	–	–	–	1754, 1730	1700, 1680
$\nu_{C=C,\ C=N\ skeletal\ ring}$	1621, 1589, 1487, 1456, 1437	1622, 1567, 1487, 1458, 1442	1655, 1626, 1564, 1487, 1456, 1434, 1422	1650, 1640, 1624,1539,1490,1450, 1420	1655, 1625, 1564, 1510, 1480, 1442, 1430, 1410
ring breathing vibration	1154, 1114	1148, 1114	1148, 1122, 1113	1050	1148
$\nu_{C\text{-}O}$	1058, 1038	1082, 1061	1082, 1038	1150, 1100	1030, 1020
ν_{Cl^-}	–	–	668 - 548	–	696-557

As the protonated form of L1 appears in complex **1**, we obtained its chloride salt (in the solid state). For comparison, the IR spectrum of HL1 (chloride salt of 2-hydroxymethyl-benzimidazole) shows a characteristic strong band at 3274 cm^{-1} assignable to $\nu_{(N\text{-}H)}$ of protonated pyridine-like nitrogen atoms of the heteroaromatic ring [17]. In turn, strong vibrations appearing at 1058 and 1038 cm^{-1} in the free ligand correspond to the stretching of $\nu_{(C\text{-}O)}$ from the hydroxymethyl group [18]; these peaks are significantly shifted in the HL1 spectrum and appear at 1082 and 1061 cm^{-1}, respectively (Table S1). In the IR spectrum of complex **1**, broad absorption bands at approximately 3602 cm^{-1} and 3337 cm^{-1} are assigned to the new peaks of $\nu_{(O\text{-}H)}$ from the hydronium cations and ethanol molecules. The weak broad peak shifted to higher wavenumbers and was observed at 3242 cm^{-1}, corresponding to $\nu_{(O\text{-}H)}$ stretching from the ligand. This result suggests that the hydroxymethyl group is engaged in H-bond formation. In turn, the presence of a strong sharp band at 3140 cm^{-1} indicates NH vibrations and shows that the nitrogen of the aromatic rings is protonated [18].

In the second synthesis (carried out in the molar ratio M: L 1: 2), the commercial ligand ($L^1$2) used undergoes the phenomenon of keto-enol tautomerism (Scheme 2A) with the formation of a more stable tautomer $L^2$2. Unexpectedly, after a few days, the first fraction in the form of greenish gold crystals was isolated. IR studies have shown that the product is $L^2$2 (melting point 267°C). The presence of bands corresponding to $\nu_{N\text{-}H}$ (3130 cm^{-1}), $\nu_{C=O(COOH)}$ (1730 cm^{-1}), $\nu_{C=O}$ (1754 cm^{-1}) and $\nu_{C\text{-}O}$ (1150, 1100 cm^{-1}) confirms this assignment. Over the next ten days, brownish red crystals of the second fraction appeared in the solution. The resulting complex **2** consists of a neutral Ru(IV) complex with chloride ions and coordinating acetonitrile molecules and a new ligand formed *in situ* (Scheme 1B). Most likely due to the presence of Ru(IV) compound, $L^2$2 is transformed to 1,4-dihydroquinoxaline-2,3-dione ($L^3$2) (Scheme 2B). This process is decarbonylation and it takes place in the -COOH group. The decarbonylation is associated with the loss of a CO molecule and forming $L^3$2. Mechanism of decarbonylation with enol-form intermediate is presented in Scheme 2B. The absence of a strong IR band at approximately 1730 cm^{-1} in the spectrum of complex **2** clearly indicates a lack of carboxylic groups and the presence of only –C=O groups. However, new bands appearing at 2250 and 2294 cm^{-1} are attributed to $\nu_{C\equiv N}$ vibrations from acetonitrile.

Table S2. Selected bond lengths (Å) and valence angles (°) for $(H_3O)_2(HL1)_2[Ru^{IV}Cl_6]\cdot 2Cl\cdot 2EtOH$.

**Bond lengths (Å)**			
Ru(1)-Cl(1)	2.3718(8)	Ru(1)-Cl(3)	2.3676(7)
Ru(1)-Cl(2)	2.3817(8)		
**Valance angles (°)**			
Cl(1)-Ru(1)-Cl(1)^{i}	180.0	Cl(2)-Ru(1)-Cl(3)	90.11(3)
Cl(2)-Ru(1)-Cl(2)^{i}	180.0	Cl(2)^{i}-Ru(1)-Cl(3)	89.89(3)
Cl(3)-Ru(1)-Cl(3)^{i}	180.0(2)	Cl(3)-Ru(1)-Cl(1)	89.97(3)
Cl(1)-Ru(1)-Cl(2)	89.93(3)	Cl(3)-Ru(1)-Cl(1)^{i}	90.03(3)
Cl(1)-Ru(1)-Cl(2)^{i}	90.07(3)		

Symmetry code: (i) -x,-y,-z.

Table S3. Hydrogen bonds for the ruthenium complexes and $L^2$2 (Å) and (°).

	D-H···A	d(D-H)	d(H···A)	d(D···A)	<(DHA)
	N(3)-H(3) ···Cl(4)^{ii}	0.93(5)	2.14(5)	2.995(4)	151(5)
	C(6)-H(6)···Cl(4)^{v}	0.93	2.71	3.620(6)	164.9
	Three-centred hydrogen bond („bifurcated”)				
	N(1)-H(1)···Cl(1)^{ii}	0.90	2.49	3.201(3)	136(5)
	N(1)-H(1)···Cl(3)^{iv}	0.90	2.74	3.379(3)	129(4)
	Four-centred hydrogen bond („trifurcated”)				
**1**	C(22)-H(22A)···Cl(1)^{vi}	0.97	2.53	3.288(3)	134.7
	C(22)-H(22A)···Cl(2)^{vii}	0.97	2.66	3.406(3)	133.8
	C(22)-H(22A)···Cl(3)^{vi}	0.97	2.79	3.501(4)	130.9
	Four-centred hydrogen bond („trifurcated”)				
	C(22)-H(22B)···Cl(1)^{viii}	0.97	2.70	3.433(3)	133.0
	C(22)-H(22B)···Cl(2)^{viii}	0.97	2.95	3.616(4)	126.8
	C(22)-H(22B)···Cl(3)^{viii}	0.97	2.49	3.302(3)	141.2
	Four-centred hydrogen bond („trifurcated”)				
	C(16)-H(16A)···Cl(1)^{ix}	0.98	2.88	3.856(3)	173.1
	C(16)-H(16B)···Cl(4)^{x}	0.98	2.90	3.503(3)	120.5
	C(16)-H(16C)···Cl(3)^{xi}	0.98	2.75	3.520(3)	136.4
	Four-centred hydrogen bond („trifurcated”)				
	C(14)-H(14C)···Cl(2)^{xii}	0.98	2.90	3.523(2)	122.6
	C(14)-H(14A)···O(12)^{xiii}	0.98	2.63	3.149(3)	113.6
**2**	C(14)-H(14B)···O(11)	0.98	2.41	3.242(3)	142.2
	C(5)-H(5)···Cl(2)^{xiv}	0.95	2.87	3.675(3)	142.9
	N(4)-H(4N)···Cl(2)^{xiv}	0.89(3)	2.54(4)	3.395(2)	161(3)
	Three-centred hydrogen bond („bifurcated”)				
	O(1)-H(1W)···O(11)	0.97(2)	2.30(1)	3.118(3)	142(1)
	O(1)-H(1W)···O(12)	0.97(2)	2.13(1)	2.857(3)	130(1)
	N(1)-H(1N)···Cl(1)^{xv}	0.72(3)	2.69(3)	3.405(2)	171(3)
	O(1)-H(2W)···Cl(1)^{xvi}	0.96(2)	2.47(5)	3.411(3)	168(2)
	N(4)-H(4N)···O(12)^{xvii}	0.88(3)	2.41(3)	3.028(3)	127(3)
**$L^2$2**	N(4)-H(4N)···N(1)^{xvii}	0.88(3)	2.17(3)	3.014(2)	161(3)
	O(13)-H(13O)···O(14)	0.88(3)	1.75(3)	2.554(2)	150(3)

Symmetry transformations used to generate equivalent atoms **(1)** (ii) x,y-1,z; (iii) x,y+1,z+1; (iv) -x,-y+1,-z+1; (v) x,y+1,z; (vi) x,y,z+1; (vii) -x,-y,-z+1; (viii) -x+1,-y,-z+1; **(2)** (ix) -x+2,y-1/2,-z+1/2; (x) -x+2,-y,-z+1; (xi) x+1,y,z; (xii) -x+1,y+1/2,-z+1/2; (xiii) x,-y+1/2,z+1/2; (xiv) -x+1,-y,-z; (xv) x-1,y,z; (xvi) x-1,-y+1/2,z-1/2; **($L^2$2)** (xvii) x+1/2,-y+3/2,z.

Table S4. Selected bond lengths (Å) and valence angles (°) for $[Ru^{IV}Cl_4(CH_3CN)_2](L^32)\cdot H_2O$.

**Bond lengths (Å)**			
Ru(1)-N(3)	2.0155(2)	Ru(1)-N(5)	2.0381(2)
Ru(1)-Cl(1)	2.3652(6)	Ru(1)-Cl(2)	2.3491(5)
Ru(1)-Cl(3)	2.3555(6)	Ru(1)-Cl(4)	2.3639(5)
N(1)-C(2)	1.346(3)	C(2)-C(3)	1.508(3)
C(3)-N(4)	1.347(3)	N(4)-C(10)	1.398(3)
C(5)-C(6)	1.382(3)	C(6)-C(7)	1.388(4)
C(7)-C(8)	1.379(4)	C(8)-C(9)	1.388(3)
C(9)-C(10)	1.393(3)	C(5)-C(10)	1.393(3)
N(1)-C(9)	1.397(3)	C(3)-O(12)	1.229(3)
C(2)-O(11)	1.226(3)		
**Valance angles (°)**			
N(3)-Ru(1)-N(5)	179.73(8)	N(3)-Ru(1)-Cl(2)	88.09(5)
N(5)-Ru(1)-Cl(2)	91.94(5)	N(3)-Ru(1)-Cl(3)	89.51(6)
N(5)-Ru(1)-Cl(3)	90.75(5)	Cl(2)-Ru(1)-Cl(3)	91.87(2)
N(3)-Ru(1)-Cl(4)	89.72(5)	N(5)-Ru(1)-Cl(4)	90.26(5)
Cl(2)-Ru(1)-Cl(4)	177.78(2)	Cl(3)-Ru(1)-Cl(4)	87.78(2)
N(3)-Ru(1)-Cl(1)	90.45(6)	N(5)-Ru(1)-Cl(1)	89.28(5)
Cl(2)-Ru(1)-Cl(1)	90.16(2)	Cl(3)-Ru(1)-Cl(1)	177.97(2)
Cl(4)-Ru(1)-Cl(1)	90.19(2)	C(3)-N(4)-C(10)	124.8(2)
C(2)-N(1)-C(9)	125.0(2)	C(2)-N(1)-H(1N)	119(3)
C(9)-N(1)-H(1N)	116(3)	C(3)-N(4)-H(4N)	117(2)
C(10)-N(4)-H(4N)	118(2)		

Table S5. Selected bond lengths (Å) and valence angles (°) for L^22.

**Bond lengths (Å)**			
N(1)-C(2)	1.298(3)	C(2)-C(3)	1.475(3)
C(3)-N(4)	1.348(3)	N(1)-C(9)	1.380(3)
N(4)-C(10)	1.377(3)	C(2)-C(11)	1.508(3)
O(13)-C(11)	1.324(3)	O(12)-C(11)	1.198(3)
C(3)-O(14)	1.244(3)	C(5)-C(6)	1.373(3)
C(6)-C(7)	1.397(3)	C(7)-C(8)	1.376(3)
C(8)-C(9)	1.400(3)	C(9)-C(10)	1.409(3)
C(5)-C(10)	1.402(3)		
**Valance angles (°)**			
C(2)-N(1)-C(9)	119.06(2)	C(3)-N(4)-C(10)	123.80(2)
N(1)-C(2)-C(3)	123.9(2)	N(1)-C(2)-C(11)	116.20(2)
C(3)-C(2)-C(11)	119.90(2)	N(1)-C(9)-C(8)	119.67(2)
N(1)-C(9)-C(10)	120.76(2)	C(8)-C(9)-C(10)	119.6(2)
N(4)-C(10)-C(5)	121.81(2)	N(4)-C(10)-C(9)	117.99(2)
C(5)-C(10)-C(9)	120.2(2)	C(7)-C(8)-C(9)	119.8(2)
C(6)-C(5)-C(10)	118.8(2)	O(12)-C(11)-O(13)	122.6(2)
O(12)-C(11)-C(2)	121.5(2)	O(13)-C(11)-C(2)	115.81(2)
O(14)-C(3)-N(4)	122.51(2)	O(14)-C(3)-C(2)	123.0(2)
N(4)-C(3)-C(2)	114.46(2)	C(5)-C(6)-C(7)	121.5(2)
C(8)-C(7)-C(6)	120.2(2)		

Table S6. UV-Vis spectroscopic data for HL1, $L^2$2 and Ru(IV) complexes.

Compound	Transitions λ, nm (ε, $dm^3 \cdot mol^{-1} \cdot cm^{-1}$)		
	**π →π*/n→π***	**LMCT π(L)→ d(Ru)**	***d – d***
HL1	209 (8512), 239 (3105), 268 (5166), 275 (4947)		
Complex **1**	226 (1325), 274 (1308), 288 (822)	360 (202), 460 (91)	590 (47)
$L^2$2	207 (8353), 230 (8753), 248 (3466), 295 (3468), 346 (3544)		
Complex **2**	218 (9450), 235 (7505), 245 (5725), 311 (5380), 325 (4654), 344 (2346)	380 (2221), 393 (2387), 453 (308)	

Table S7. Electrochemical data (in V *vs* Ag/AgCl) for the ruthenium complexes obtained by cyclic voltammetry (CV) on GCE and by differential pulse voltammetry (DPV) on CF disk microelectrode.

Complex	Day	CV				DPV	
		E_{pa}	E_{pc}	ΔE_p	$E_{1/2}$	E_{pc}	$W_{1/2}$
		Ru(IV)/Ru(III)[b→a]					
**1**	1st	0.128	-0.004	0.124	0.062	-0.008	0.096
	2nd - 7th	0.133	0.070	0.063	0.102	0.110	0.090
		Ru(III)/Ru(II)[b→a]					
	1st	-0.162	-0.308	#	#	-0.162	0.126
	2nd - 7th	-0.153	-0.210	0.057	-0.182	-0.194	0.088
		Ru(II)/Ru(I)[b→a]					
	1st	-0.162	-0.308	#	#	-0.323	0.134
	2nd - 7th	-0.355	-0.416	0.061	-0.386	-0.392	0.088
**2**		Ru(IV)/Ru(III)[a]					
	1st	0.169	0.109	0.060	0.139	0.150	0.091
		Ru(III)/Ru(II)*+ Ru(II)/Ru(I)*					
	1st	~-0.130	~-0.180	~0.05	~-0.16	~-0.25	#

Conditions: 0.1 M $TBAPF_6$ in mixed solvent; CH_3CN/EtOH (3 : 2, *v/v*); CV: GCE (∅ = 2 mm), scan rate 0.1 V s^{-1}; DPV: CF (∅ = 33 µm), pulse amplitude of 20 mV, pulse width 80 ms, scan rate 0.02 V s^{-1}. [a]Reversible couple. [b]Irreversible couple. *Indefinite couple. # – not determinable, $E_{1/2} = ½(E_{pc} + E_{pa})$, $\Delta E_p = E_{pa} – E_{pc}$, E_{pa}, E_{pc} – anodic, cathodic peak potential, respectively, $W_{1/2}$ – the width of the DPV peak at half-height.

Table S8. Bacteriostatic activities of the investigated ruthenium complexes, ruthenium salt and ligands as MIC concentrations, expressed in mM and μg/ml.

Compound	BACTERIA							
	S. aureus		***E. coli***		***P. aeruginosa PAO1***		***P. aeruginosa LES B58***	
	mM	μg/ml	mM	μg/ml	mM	μg/ml	mM	μg/ml
$RuCl_3 \cdot xH_2O$	> 1	> 207	> 1	> 207	> 1	>207	> 1	>207
L1	> 1	> 148	> 1	> 148	> 1	> 148	> 1	> 148
L^22	> 1	> 190	> 1	> 190	> 1	> 190	> 1	> 190
**1**	1	813	1	813	1	813	1	813
**2**	1	505	> 1	> 505	> 1	> 505	> 1	> 505
Streptomycin	0.0625	36	0.125	73	0.0625	36	0.5	291

L1 – 2-hydroxymethylbenzimidazole, L^22 – 3-oxo-4H-quinoxaline-2-carboxylic acid, $(H_3O)_2(HL1)_2[Ru^{IV}Cl_6]\cdot 2Cl\cdot 2EtOH$ (**1**), $[Ru^{IV}Cl_4(CH_3CN)_2](L^32)\cdot H_2O$ (**2**)

Table S9. Crystal data and structure refinements for **1**, **2** and $L^2$2.

	**1**	**2**	**$L^2$2**
Empirical formula	$C_{20}H_{30}Cl_8N_4O_6Ru$	$C_{12}H_{14}Cl_4N_4O_3Ru$	$C_9H_6N_2O_3$
Formula weight	807.15	505.14	190.16
Temperature (K)	293(2)	120(2)	120(1)
Wavelength (Å)	0.71073	0.7107	0.7107
Crystal system, space group	triclinic, $P\ \bar{1}$	monoclinic, $P\ 2_1/c$	orthorhombic, $P\ n\ a\ 2_1$
Unit cell dimensions			
a (Å)	7.1050(2)	7.02740(1)	11.3958(3)
b (Å)	9.4880(2)	15.2807(2)	11.8852(3)
c (Å)	13.4130(3)	17.2332(3)	6.0131(2)
α (˚)	81.977(2)		
β (˚)	77.0380(1)	98.376(2)	
γ (˚)	71.6460(1)		
Cell volume (Å^3)	834.01(3)	1830.82(5)	814.42(4)
Z, Calculated density (g cm^{-3})	1, 1.607	4, 1.833	4, 1.551
Absorption coefficient (mm^{-1})	1.149	1.458	0.120
F(000)	406	1000	392
Crystal size (mm)	0.20 × 0.10 × 0.01	0.350 × 0.230 × 0.040	0.360 × 0.300 × 0.100
Theta range for data collection (°)	2.85-27.46	2.92-34.57	3.43 to 31.87
Limiting indices	$-8 \le h \le 9$, $-12 \le k \le 12$, $-17 \le l \le 17$	$-11 \le h \le 11$, $-24 \le k \le 24$, $-27 \le l \le 27$	$-16 \le h \le 16$, $-17 \le k \le 17$, $-8 \le l \le 8$
Reflections collected/unique/observed [$I > 2$sigma(I)]	6756/3780 [R_{int} = 0.0186]	113598/7649 [R_{int} =0.0660]	16987/2674 [R_{int} =0.0444]
Completeness to 2θ (%)	2θ = 25.24°, 99.3	2θ = 25.24°, 99.9	2θ = 25.24°, 99.9
Absorption correction		Semi-empirical from equivalents	
Maximum and minimum transmission	0.9886 and 0.8027	1.000 and 0.725	1.00000 and 0.56715
Refinement method		Full-matrix least-squares on F^2	
Data/restraints/parameters	3780/0/188	7649/3/235	2674/1/135
Goodness-of-fit on F^2	1.005	1.136	1.065
Final R indices [I >2sigma(I)]	R_1 = 0.0436, wR_2 = 0.1232	R_1 = 0.00333, wR_2 = 0.0684	R_1 = 0.00430, wR_2 = 0.0980
R indices (all data)	R_1 = 0.0462, wR_2 = 0.1262	R_1 = 0.0542, wR_2 = 0.0810	R_1 = 0.0587, wR_2 = 0.1074
Largest differences in peak and hole (e Å^3)	0.859 and -1.257	1.221 and -0.678	0.449 and -0.237

3. Supporting figures

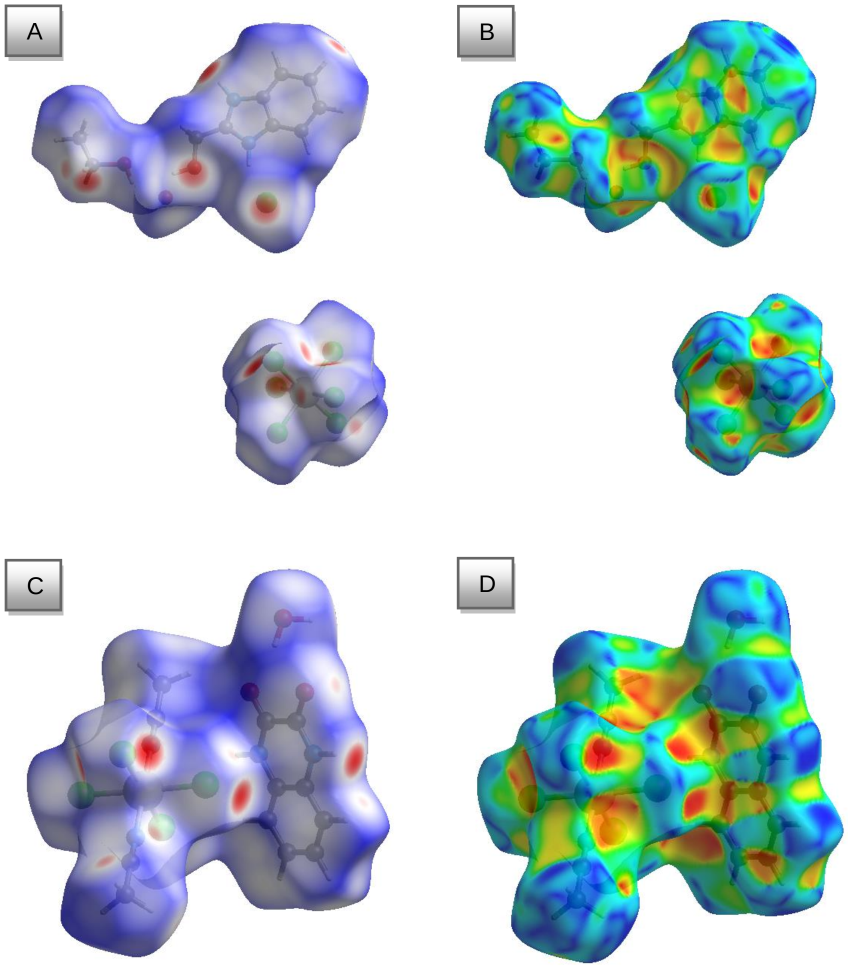

Figure S1. The Hirshfeld surfaces of complexes **1** (A) and **2** (C) mapped with 3D d_{norm} (with transparency enabled) and shape index functions (B and C).

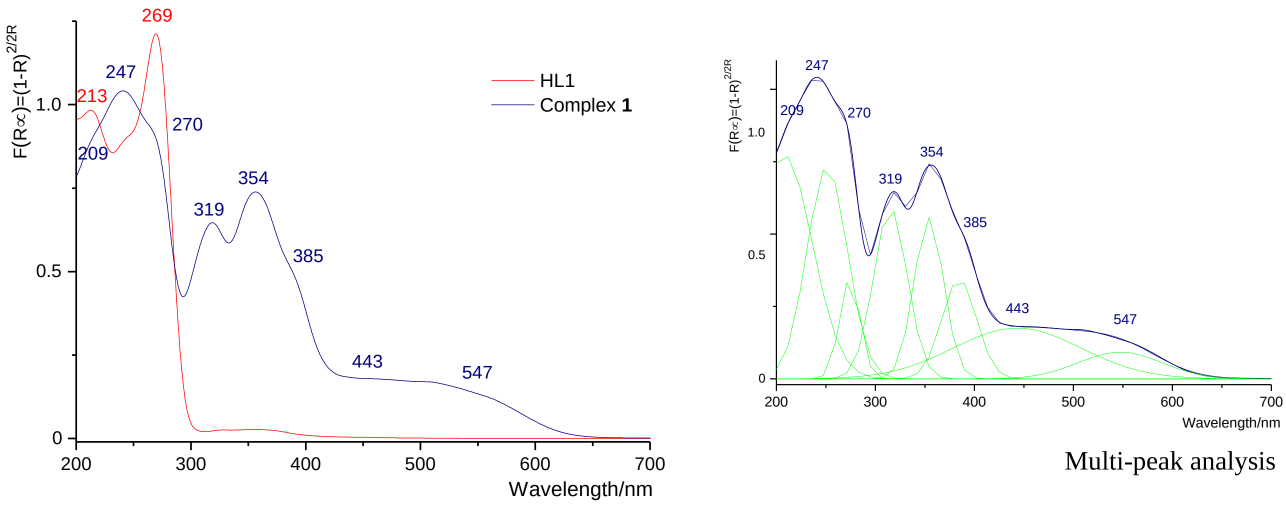

A

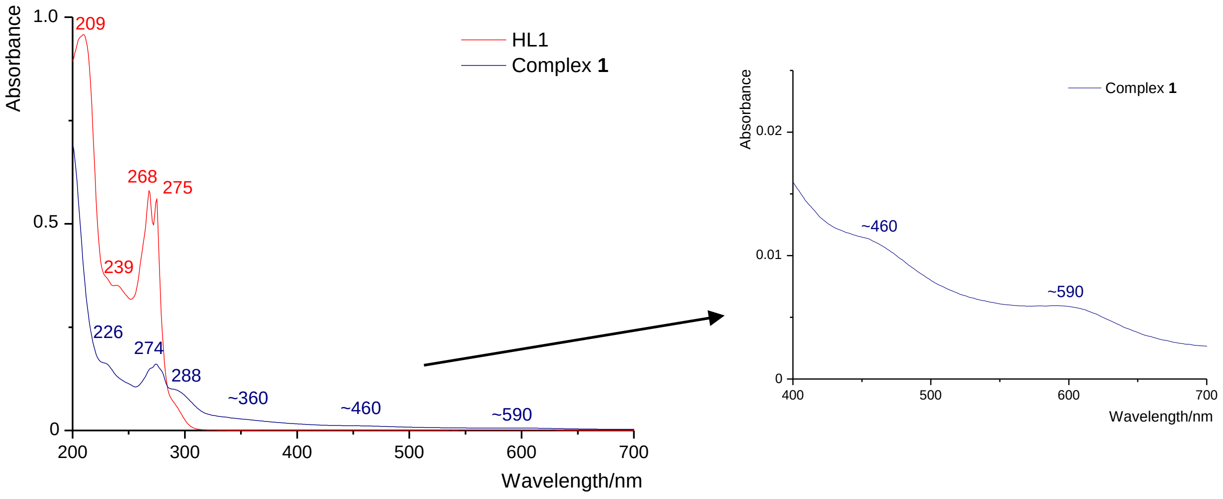

B

Figure S2. UV-Vis absorption spectra of solid state (A) and in aqueous solution (B) for complex **1** and HL1.

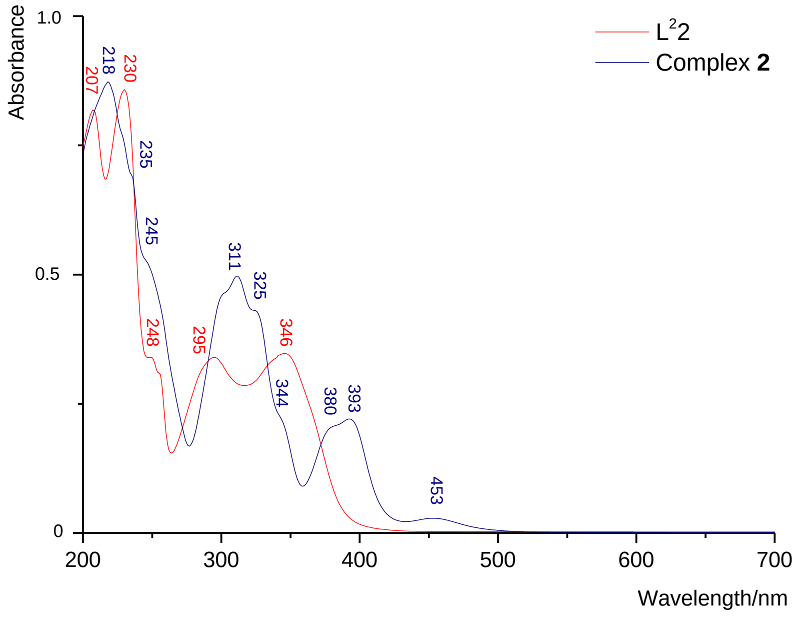

Figure S3. UV-Vis absorption spectra in aqueous solution for complex **2** and L^22.

The diffuse reflectance spectrum of HL1 is shown in Figure S2A. In the UV region, the high-intensity bands observed at approximately 213 and 269 nm in the benzimidazole derivative are due to intra-ligand transitions (π–π*) [19]. In the reflectance spectrum of complex **1** (Figure S2A), the relatively intense bands at approximately 209, 247 and 270 nm can be assigned to intra/inter-ligand transitions from the heteroaromatic moieties, whereas moderately intense absorption bands in the and visible part of the spectrum (354, 385 and 443 nm) are attributed to ligand–to–metal charge transfer (LMCT) from the chloride ion to the metal centre. The lower intensity broad band in the 500-600 nm region is probably assigned to the *d*–*d* transitions (~547 nm) for ruthenium(IV) due to the state of the d^4 ion.

The data obtained for the reflectance spectrum of complex **1** are in agreement with the experimental data in aqueous solution. The electronic spectrum of HL1 displayed two broad absorption bands (Figure S2B, Table S6) that can be assigned to π→π* transitions in the delocalized π–electron system [20]. The split bands at 268 and 275 nm appear as doublets due to the probable existence of a tautomeric structure [21], as supported by comparing our spectrum with that of benzimidazole derivatives [22]. The electronic spectrum of complex **1** displayed three distinct absorption bands in water (Figure S2B). The bands at 226, 274 and 288 nm may be assigned to the low-energy π→π* transitions within the benzimidazole moieties [19]. The UV-Vis spectrum is also characterized by a band at ~360 nm and by a second weaker band at ~460 nm (Table S6, Figure S2B). The former bands can be ascribed to $\pi Cl \rightarrow t_{2g}Ru$ LMCT (Ru-Cl) transitions involving the four coplanar chlorides [23]. The last absorption band in the visible part of the spectrum with a maximum at ~590 nm is attributed to the *d*–*d* transition. According to the Tanabe–Sugano diagram, the *d*–*d* transition has been assigned to the $^3T_{1g} \rightarrow {}^3E_g$ transition for low-spin Ru(IV) complex ($t_{2g}{}^4e_g{}^0$ configuration) in an O_h environment.

More complicated electronic spectra were recorded for the ligand $L^2$2 and complex **2** (Figure S3). Analysis of the UV-Vis spectrum of ligand $L^2$2 indicated that strong or moderate intensity bands in the 200 – 390 nm region are related to the intra-ligand π→π*/n→π* transitions. It is noteworthy that in the process of complexation, the ligand was transformed into a diketone, and the bands corresponding to the n→π* transitions are in the 280 – 350 nm region in the spectrum of compound **2**. The next bands (380, 393, 453 nm) in the electronic spectrum of the complex were assigned to CT transitions (π(L)→d(Ru)).

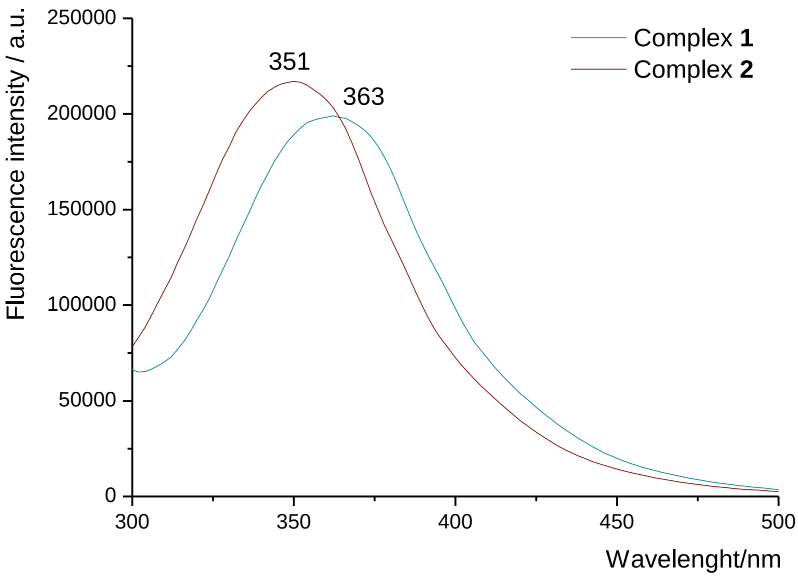

Figure S4. Emission spectra of ruthenium complexes in an aqueous solution.

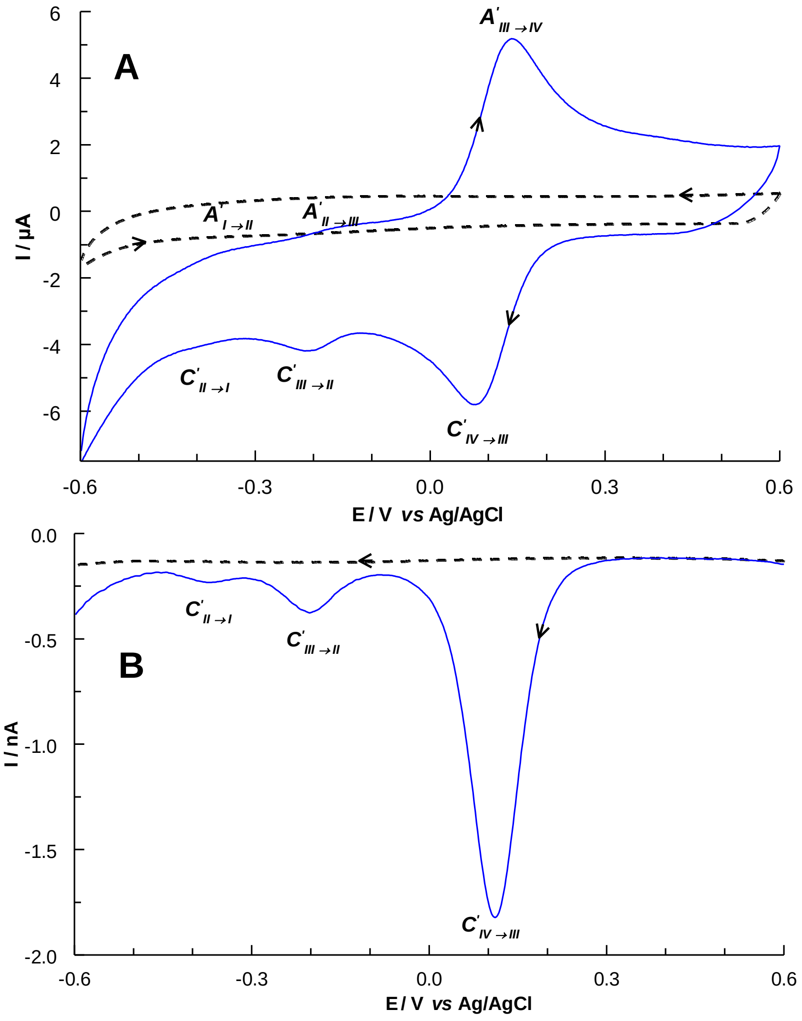

Figure S5. CV (A) and DPV (B) voltammograms of 1mM complex **1** recorded on the last day of the investigations. Conditions as shown in Figure 7.

4. Supporting references

1. Jabłońska-Wawrzycka, A.; Rogala, P.; Michałkiewicz, S.; Hodorowicz, M.; Barszcz, B. Ruthenium complexes in different oxidation states: synthesis, crystal structure, spectra and redox properties. *Dalton Trans.* **2013**, *42*, 6092–6101, doi:10.1039/c3dt32214a.
2. Kubelka, P.; Munk, F. Ein beitrag zur optik der farbanstriche. *Z. Tech. Phys.* **1931**, *12*, 593–601.
3. Kahn, O. *Molecular Magnetism*; Wiley-VCH: New York, 1993; Vol. 6; ISBN 9780471188384.
4. Rogala, P.; Czerwonka, G.; Michałkiewicz, S.; Hodorowicz, M.; Barszcz, B.; Jabłońska-Wawrzycka, A. Synthesis, structural characterization and antimicrobial evaluation of ruthenium complexes with heteroaromatic carboxylic acids. *Chem. Biodiversity* **2019**, *16*, e1900403, doi:10.1002/cbdv.201900403.
5. Blanc, R.; González-Casado, A.; Navalón, A.; Vílchez, J.L. On the estimate of blanks in differential pulse voltammetric techniques: application to detection limits evaluation as recommended by IUPAC. *Anal. Chim. Acta* **2000**, *403*, 117–123, doi:10.1016/S0003-2670(99)00569-3.
6. Nonuis, B. V. Nonius COLLECT, Delft, The Netherlands 1997–2000.
7. Otwinowski, Z.; Minor, W. Processing of X-ray diffraction data collected in oscillation mode. *Methods Enzymol.* **1997**, *276*, 307–326, doi:10.1016/S0076-6879(97)76066-X.
8. Altomare, A.; Cascarano, G.; Giacovazzo, C.; Guagliardi, A.; Burla, M.C.; Polidori, G.; Camalli, M. SIR92 – a program for automatic solution of crystal structures by direct methods. *J. Appl. Crystallogr.* **1994**, *27*, 435, doi:10.1107/S002188989400021X.
9. Sheldrick, G.M. SHELX2014/SHELX2017, Programs for crystal structure determination, Universität Göttingen, Germany 2017.
10. Sheldrick, G.M. Crystal structure refinement with SHELXL. *Acta Crystallogr. Sect. C Struct. Chem.* **2015**, *C71*, 3–8, doi:10.1107/S2053229614024218.
11. Rigaku Oxford Diffraction. CrysAlis PRO. Rigaku Oxford Diffraction, Yarnton, England 2015.
12. Brandenburg, K.; Putz, H. Diamond - Crystal and Molecular Structure Visualization Crystal Impact. Rathausgasse 30, D-53111 Bonn, GbR, version 3.1 2000.
13. Marciniak, H.; Diduszko, R. XRAYAN - X-ray Phase Analysis, Warszawa, version 2.9 1994.
14. Wolff, S.K.; Grimwood, D.J.; McKinnon, J.J.; Turner, M.J.; Jayatilaka, D.; Spackman, M.A. Crystal Explorer, University of Western Australia, Perth, Australia 2013.
15. Spackman, M.A.; Jayatilaka, D. Hirshfeld surface analysis. *CrystEngComm* **2009**, *11*, 19–32, doi:10.1039/B818330A.
16. Merritt, J.H.; Kadouri, D.E.; O'Toole, G.A. Growing and analyzing static biofilms. In *Current Protocols in Microbiology*; John Wiley & Sons, Inc.: Hoboken, NJ, USA, 2005; Vol. Chapter 1, p. Unit 1B.1.
17. Morgan, K.J. The infrared spectra of some simple benzimidazoles. *J. Chem. Soc.* **1961**, *455*, 2343–2347, doi:10.1039/jr9610002343.
18. Nakamoto, K. *Infrared and Raman spectra of inorganic and coordination compounds: Part B: Applications in coordination, organometallic, and bioinorganic chemistry*; 6th ed.; John Wiley & Sons: New Jersey, 2009; ISBN 9780471744931.
19. Lever, A.B.P. *Inorganic electronic spectroscopy*; 2nd ed.; Elsevier: Amsterdam, 1984;
20. Araya-Hernández, C.G.; Morales, R.G.E. Sulfur aromatic heterocycles: a new kind of solar ultraviolet-B radiation actinometers. *J. Photochem. Photobiol. A* **2006**, *177*, 125–128, doi:10.1016/j.jphotochem.2005.03.029.
21. Issa, R.M.; El-Daly, S.A.; El-Wakiel, N.A. UV/Vis, IR and 1H NMR spectroscopic studies of bisazo-dianil compounds based on 5-(2-carboxyphenyl azo)-salicylaldehyde and primary diamines. *Spectrochim. Acta Part A* **2003**, *59*, 723–728, doi:10.1016/S1386-1425(02)00218-

4.
22. Krishnamurthy, M.; Phaniraj, P.; Dogra, S.K. Absorptiometric and fluorimetric study of solvent dependence and prototropism of benzimidazole homologues. *J. Chem. Soc. Perkin Trans. 2* **1986**, 1917–1925, doi:10.1039/p29860001917.
23. Duff, C.M.; Heath, G.A. From [RuX_6] to [$Ru(RCN)_6$]: synthesis of mixed halide–nitrile complexes of ruthenium, and their spectroelectrochemical characterization in multiple oxidation states. *J. Chem. Soc., Dalton. Trans.* **1991**, 2401–2411, doi:10.1039/DT9910002401.
